# A study on the AMACR catalysed elimination reaction and its application to inhibitor testing†
†Electronic supplementary information (ESI) available: ^1^H NMR spectra of synthesised compounds; details of X-ray crystal structure determination of compound **35**; original data for Table 1; plots of fluorescence resulting from reaction of sensors **33** and **34** with fluoride solutions. CCDC 1408401. For ESI and crystallographic data in CIF or other electronic format see DOI: 10.1039/c5ob01541c



**DOI:** 10.1039/c5ob01541c

**Published:** 2015-11-05

**Authors:** Maksims Yevglevskis, Guat L. Lee, Jenny Sun, Shiyi Zhou, Xiaolong Sun, Gabriele Kociok-Köhn, Tony D. James, Timothy J. Woodman, Matthew D. Lloyd

**Affiliations:** a Medicinal Chemistry , Department of Pharmacy & Pharmacology , University of Bath , Claverton Down , Bath BA2 7AY , UK . Email: M.D.Lloyd@bath.ac.uk ; Fax: +44 (0)1225 386114; b Department of Pharmacy , Shandong University , People's Republic of China; c Department of Chemistry , University of Bath , Claverton Down , Bath BA2 7AY , UK

## Abstract

α-Methylacyl-CoA racemase (AMACR; P504S) catalyses a key step in the degradation of branched-chain fatty acids and is important for the pharmacological activation of Ibuprofen and related drugs. Levels of AMACR are increased in prostate and other cancers, and it is a drug target. Development of AMACR as a drug target is hampered by lack of a convenient assay. AMACR irreversibly catalyses the elimination of HF from 3-fluoro-2-methylacyl-CoA substrates, and this reaction was investigated for use as an assay. Several known inhibitors and alternative substrates reduced conversion of 3-fluoro-2-methyldecanoyl-CoA by AMACR, as determined by ^1^H NMR. The greatest reduction of activity was observed with known potent inhibitors. A series of novel acyl-CoA esters with aromatic side chains were synthesised for testing as chromophoric substrates. These acyl-CoA esters were converted to unsaturated products by AMACR, but their use was limited by non-enzymatic elimination. Fluoride sensors were also investigated as a method of quantifying released fluoride and thus AMACR activity. These sensors generally suffered from high background signal and lacked reproducibility under the assay conditions. In summary, the elimination reaction can be used to characterise inhibitors, but it was not possible to develop a convenient colorimetric or fluorescent assay using 3-fluoro-2-methylacyl-CoA substrates.

## Abstract 2

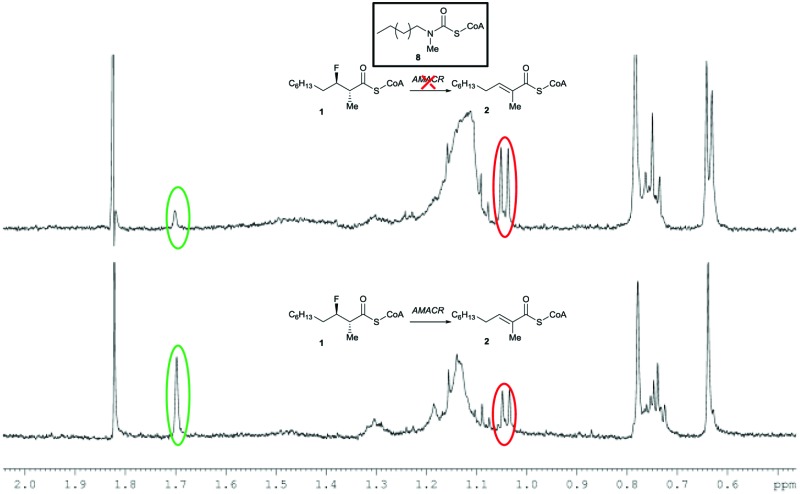
The elimination of fluoride from 3-fluoro-2-methylacyl-CoA substrates by α-methylacyl-CoA racemase (AMACR 1A; P504S) was investigated as a method for determining enzyme activity and inhibitor potency.

## Introduction

α-Methylacyl-CoA racemase (AMACR, P504S; E.C. 5.1.99.4) catalyses a key step in the degradation of branched-chain fatty acids.^1–3^ The enzyme catalyses the conversion of either epimer of a 2-methylacyl-CoA ester into a *ca.* 1 : 1 mixture of 2*R*- and 2*S*-epimers.^4,5^ β-Oxidation of 2-methylacyl-CoAs requires 2*S*-configuration,^6,7^ but both *R*- and *S*-2-methylacyl-CoAs are produced *in vivo* and are derived from dietary fatty acids.^3^ Thus, AMACR enables metabolism of *R*-2-methylacyl-CoAs. It is also important in the pharmacological activation of *R*-Ibuprofen and related drugs.^3,5,8^ AMACR has also been proposed to be involved in the uni-directional chiral inversion of mandelic acid in mammals^9^ but this was recently disproved.^10^

AMACR protein levels and enzyme activity are increased in prostate cancers,^11,12^ myxofibrosarcomas,^13^ a subset of colon cancers^14^ and various other cancers^8^ and it is widely recognised as a promising drug target.^3,8,15–18^ Genetic knock-down of AMACR reduces proliferation of cultured cancer cells^13,15,16,19^ and restores androgen-dependent growth in some prostate cancer cell lines.^16^ Relatively few chemical inhibitors of AMACR have been reported,^17–20^ largely due to the lack of a convenient, high-throughput assay. Current assay methods include wash-out of tritium from a labelled substrate followed by measurement of radioactive water^1,2,19^ or wash-in of deuterium from D_2_O followed by ^1^H NMR analyses.^4,5,10^ These assays are probably subject to a kinetic isotope effect, and are low-throughput and labour-intensive. In addition, the ^1^H NMR assay also suffers from signal overlap of the substrate/product 2-methyl group, thus making it more difficult to quantify activity. Characterisation of AMACR inhibitors using HPLC assays^17,18^ has also been reported, but these assays are also low throughput. Despite these difficulties, one of the identified inhibitor pro-drugs, trifluoroibuprofen, shows promising anti-prostate cancer effects in *in vivo* models.^21^

Other approaches have also been used to develop a convenient assay for AMACR activity. The use of acyl-CoA oxidase as a coupling enzyme enables a colorimetric assay to be performed.^22^ This enzyme is not commercially available and rationally designed acyl-CoA inhibitors of AMACR are also likely to inhibit the coupling enzyme, complicating the analysis. Coupled enzyme assays for other racemases/epimerases have also been reported,^23–27^ but these are not readily adaptable to measuring AMACR activity.

Direct measurement of racemisation by MCR (the bacterial homologue of AMACR from *M. tuberculosis*) using circular dichroism has been reported,^28^ but this was not used for inhibitor testing. Assays for several other racemases/epimerases using circular dichroism^27,29–31^ or polarimetry^32–34^ have been developed, but these are generally low-throughput. Moreover, acyl-CoA inhibitors with aromatic side-chains are likely to undergo racemisation and this will complicate the determination of inhibitor properties. Therefore, these assays have serious limitations when determining inhibitor potency.

**Scheme 1:**
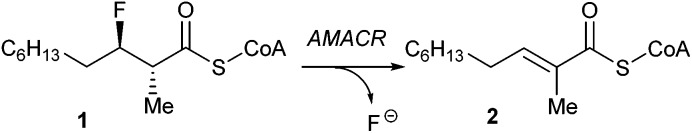
The elimination reaction catalysed by AMACR.

It has recently been reported that AMACR performs an elimination reaction in which HF is eliminated from 3-fluoro-2-methylacyl-CoAs (such as **1**) to give unsaturated acyl-CoAs (such as **2**) (Scheme 1).^35^ This reaction is irreversible which is an advantage compared to assays using isotopic labels^1,2,4,5,10,19^ or ‘racemisation’^17,18^ as their reversibility makes them more difficult to interpret. In addition, the 2-methyl peaks of **1** and **2** are non-overlapping in the ^1^H NMR spectrum, simplifying the measurement of substrate conversion.

The elimination reaction also offers the possibility of translation into a convenient, colorimetric or fluorometric assay by manipulation of the substrate side-chain or by the use of fluoride sensors. Assays using fluoride-specific electrodes to measure enzyme activity have also been reported,^36–38^ but these are generally low-throughput, require relatively large volumes and are not easy to adapt to a microtitre plate format.^39^ A number of highly sensitive molecular fluoride sensors have been reported in the literature, which give an increase in absorbance or fluorescence upon reaction with fluoride. However, there are relatively few that can be used in aqueous buffers.^40–43^ The development of a convenient high-throughput assay is essential for the development of AMACR as a drug target.

In this paper, the use of the AMACR-catalysed fluoride elimination reaction for the characterisation of inhibitors is investigated. Reduction in enzyme activity in the presence of other known AMACR substrates and inhibitors was observed by ^1^H NMR. The use of 3-fluoro-2-methylacyl-CoA substrates with aromatic side-chains and fluoride sensors in order to translate this reaction into a colorimetric or fluorescent assay format is also investigated.

## Results and discussion

Use of the elimination assay for inhibitor characterisation was initially performed by incubation of recombinant human AMACR 1A^4^ with a series of known ‘inhibitors’ (Table 1) and substrate **1**. The chosen ‘inhibitors’ included the known AMACR substrates^5^ Fenoprofenoyl-CoA **3**, Flurbiprofenoyl-CoA **4**, Ibuprofenoyl-CoA **5**, Ketoprofenoyl-CoA **6**, and Naproxenoyl-CoA **7**. These are expected to behave as competitive inhibitors. Also chosen was *N*-dodecyl-*N*-methyl-carbamoyl-CoA **8**, a transition state analogue and the most potent AMACR inhibitor described to date.^18^ Ebselen **9**, Ebselen oxide **10** and Rose Bengal **11** were also chosen for study as these are reported to be good inhibitors of human AMACR 1A.^19^ Enzyme was pre-incubated with inhibitor for 10 min. to allow binding before addition of substrate at 100 μM final concentration. After 1 h the assay was terminated and the level of substrate conversion was determined by ^1^H NMR. Control experiments showed that the enzyme was fully active^35^ in the presence of 1 mM fluoride solution, indicating that any reduction in activity was due to the presence of the ‘inhibitor’.

**Table 1:** Inhibition of (2*R*,3*R*)-3-fluoro-2-methyldecanoyl-CoA **1** conversion by AMACR in the presence of known substrates and inhibitors. Compound numbers for inhibitors refer to structures shown below. Conversions are means of two replicate readings ± standard deviations of the sample and are normalised to positive controls [(substrate **1** conversion in presence of inhibitor/substrate **1** conversion in absence of inhibitor) × 100]. Positive controls lacking inhibitor converted *ca.* 50% of substrate **1** after 1 h incubation. See ESI Table 1 for absolute substrate conversion levels in the presence of inhibitors and positive controls

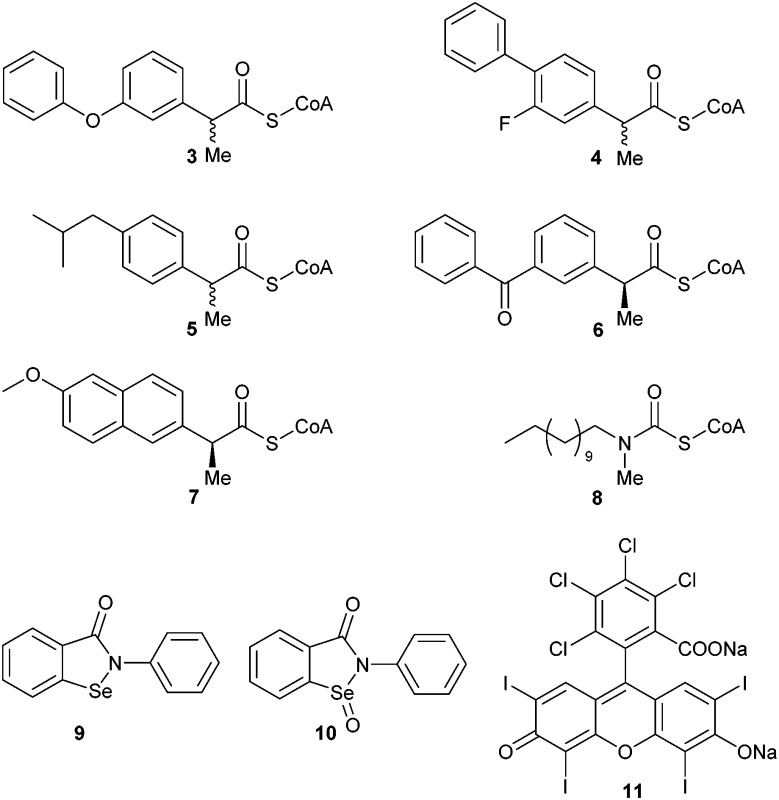		
Inhibitory compound	Relative conversion in presence of inhibitor	Relative reduction compared to no inhibitor
None	100%	0%
**3**	74.9 ± 0.4%	25.1%
**4**	91.6 ± 2.0%	8.4%
**5**	74.1 ± 7.2%	25.9%
**6**	92.0 ± 8.6%	8.0%
**7**	88.8 ± 4.0%	11.2%
**8**	16.8 ± 1.8%	83.2%
**9**	<5%	>95%
**10**	88.2 ± 5.4%	11.8%
**11**	<5%	>95%

**Fig. 1:**
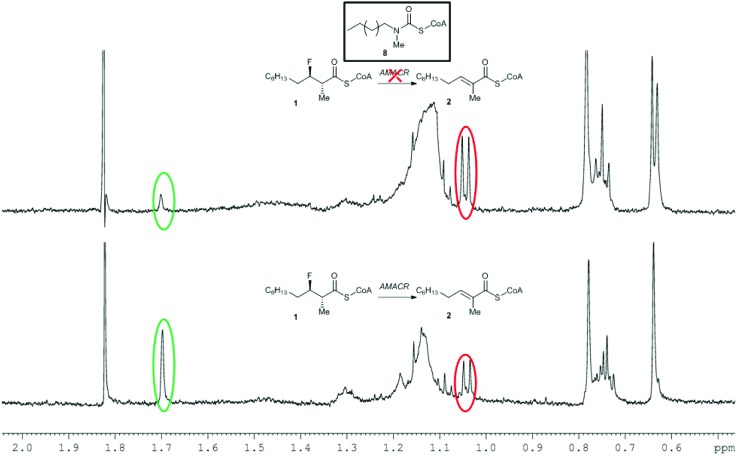
Reduction of conversion of **1** to **2** in the presence of inhibitor **8**. Above, ^1^H NMR spectrum following incubation with active AMACR in the presence of **8**; below, ^1^H NMR spectrum of positive control containing active AMACR in the absence of inhibitor. Red and green circles show methyl group signals for substrate **1** and unsaturated product **2**, respectively.

In the absence of inhibitor, *ca.* 50% of substrate **1** was converted into unsaturated product **2** by active AMACR. Negative controls containing heat-inactivated enzyme showed <5% conversion of **1** to **2**, levels of which did not change over the incubation period. The presence of each ‘inhibitor’ (at 100 μM final concentration) resulted in a reduction in the level of conversion of **1** (Table 1). Compounds **3–7** showed moderate levels of inhibition in most cases, with the most significant reduction in activity occurring with Fenoprofenoyl-CoA **3** and Ibuprofenoyl-CoA **5**. Modest levels of inhibition are expected with compounds **3–7**, as the concentration of substrate **1** (100 μM) is significantly above its reported *K*_m_ value (21 μM).^35^ These high substrate concentrations will reduce the apparent effect of competitive inhibitors, but relative high concentrations of **1** are required to perform the ^1^H NMR analyses. Incubation of the known highly potent AMACR inhibitor **8** ^18^ resulted in a very significant reduction in activity (Fig. 1), consistent with it being a good inhibitor (reported IC_50_ value of 98 nM^18^). Ebselen **9** and Rose Bengal **11** were also highly potent under the assay conditions, with no detectable conversion of **1**. In contrast, Ebselen oxide **10** was a modest inhibitor, having a similar potency to the alternative substrates **3–7**. This result was surprising as Wilson *et al.* ^19^ report that **10** was their most potent inhibitor (IC_50_ value of 790 nM), compared to **9** (IC_50_ value of 10 μM) and **11** (IC_50_ value of 2.8 μM). Ebselen **9** is reported to be an irreversible inhibitor of AMACR,^19^ and IC_50_ values are an inappropriate measure of potency as inhibition levels are dependent on the rate of inactivation. Rose Bengal **11** is a non-specific inhibitor of a number of enzymes, and inhibition appears to be related to generation of reactive oxygen species upon irradiation with UV light.^44–46^ The differences in observed behaviour between the two studies probably results from the different modes of action for **9**, **10** and **11**, meaning that the results of the two studies are not directly comparable.

Although AMACR inhibitor testing using this method offers a number of advantages, including irreversible formation of the product and non-overlapping signals for substrate **1** and product **2**, the method is still low-throughput as ^1^H NMR is used to quantify conversion levels. Translation of this method to a colorimetric or fluorescent assay is therefore desirable.

**Scheme 2:**
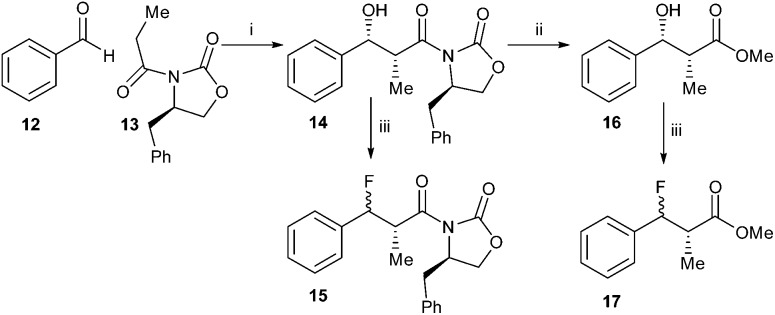
Synthesis of **15** and **17**. Reagents and conditions: i: Bu_2_BOTf, i-Pr_2_EtN, DCM, –78 °C, 99%; ii: NaOMe, MeOH, 0 °C, 36%; iii. DAST, DCM, –78 °C. Stereochemical course of reaction iii: **14** to **15**, 74%, 53% de; **16** to **17**, 53%, 50% de.

In order to do this, synthesis of acyl-CoA substrates with aromatic side-chains was investigated as it was anticipated that the unsaturated product would absorb in the visible spectrum. Synthesis of the *anti*-substrates was desired, as *syn*-substrates are prone to undergo non-enzymatic elimination.^35^ In the first synthesis, benzaldehyde **12** and the *R*-Evan's auxiliary protected propanoic acid **13** were condensed to give alcohol **14** (Scheme 2). However, treatment of **14** with DAST resulted in loss of stereochemistry upon introduction of the fluoride to give a mixture of diastereomers of **15**. For aliphatic side-chains, the replacement of the 3-hydroxy group with fluoride proceeds *via* an S_N_2 mechanism with inversion of stereochemistry. This loss of stereochemistry is probably due to an S_N_1 reaction occurring, with consequent addition of fluoride to both faces of the stabilised benzylic carbocation. Conversion of **14** to the methyl ester **16** followed by treatment with DAST also resulted in significant loss of stereochemistry on conversion to **17**, suggesting that steric hindrance by the chiral auxiliary was not the deciding factor.

**Scheme 3:**
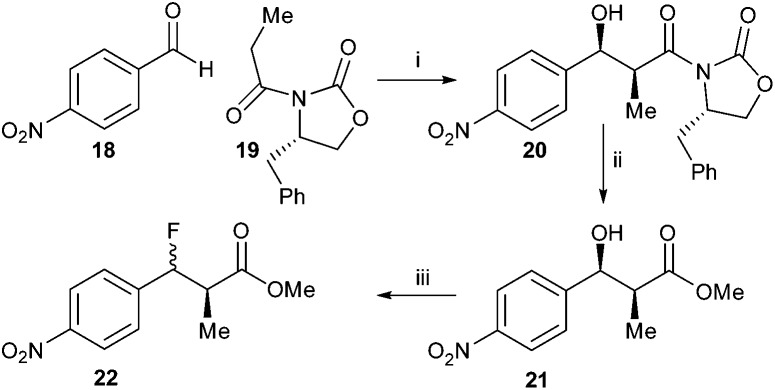
Synthesis of **22**. Reagents and conditions: MgCl_2_, TMSCl, i-Pr_2_EtN, EtOAc, rt, then TFA : MeOH 1 : 9, rt, 71%; ii: NaOMe, MeOH, 0 °C, 53%; iii: DAST, DCM, –78 °C. 69% (68% de).

Synthesis of the 4-nitrophenyl- derivative was investigated (Scheme 3) in order to destabilise the carbocation intermediate and hence improve diastereoselectivity. Condensation of 4-nitrobenzaldehyde **18** with *S*-Evan's auxiliary protected propanoic acid **19** gave **20**, which was converted to methyl ester **21**. However, treatment with DAST still resulted in a mixture of *syn*- and *anti*-**22** (68% de). The diastereomeric selectivity was somewhat improved compared to conversion of **16** to **17**, suggesting that a carbocation intermediate had been destabilised and the S_N_2 reaction was now more favoured.

**Scheme 4:**
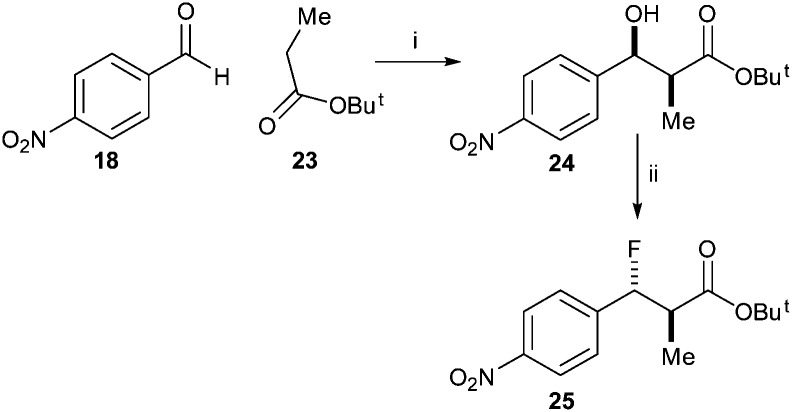
Synthesis of *tert*-butyl 3-fluoro-2-methyl-3-(4-nitrophenyl)propanoate **25** Reagents and conditions: i. LDA, THF, –78 °C, 52%; ii. DAST, DCM, –78 °C, 51%.

In contrast, condensation of *tert*-butyl-protected propanoic acid **23** with 4-nitrobenzaldehyde **18** gave **24** as a pair of enantiomers. Treatment with DAST gave the desired ester **25** (Scheme 4).

**Scheme 5:**
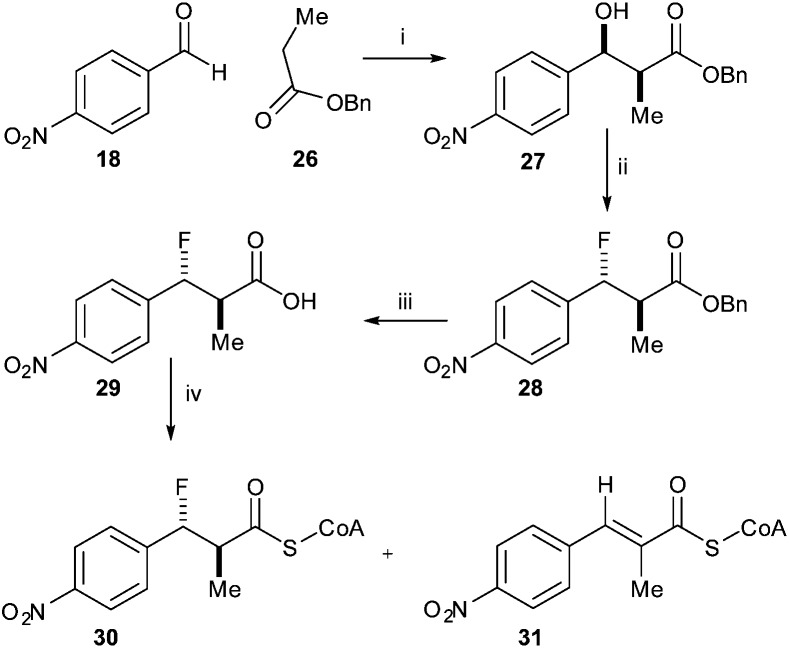
Synthesis of *anti*-3-fluoro-2-methyl-3-(4-nitrophenyl)propanoyl-CoA **30**. Reagents and conditions: i: LDA, THF, –78 °C, separation of *syn*- and *anti*-isomers; 17% yield for *syn*-isomer; ii: DAST, DCM, –78 °C, 42%; iii: TMSI, CHCl_3_, 40 °C, 90%; iv: CDI, DCM, rt, then CoA-Li_3_, 0.1 M NaHCO_3_ aq., THF, rt.

Similarly, reaction of benzyl-protected propanoic acid **26** with 4-nitrobenzaldehyde **18** gave **27**. Treatment with DAST gave the desired *anti*-product **28** (Scheme 5). Removal of the benzyl protecting group with TMSI gave acid **29**. However, conversion of the acid to the acyl-CoA ester **30** using CDI resulted in formation of a significant amount of the eliminated acyl-CoA ester **31**, the expected enzymatic product. This substrate and product mixture could not be easily separated. The elimination of **30** to give **31** is probably driven by the thermodynamic stability of the conjugated product.

**Fig. 2:**
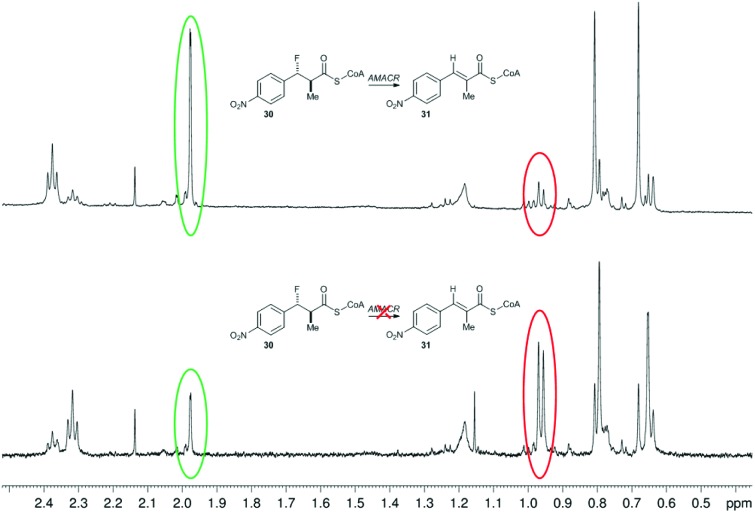
Conversion of 3-fluoro-2-methyl-3-(4-nitrophenyl)propanoyl-CoA **30** by human AMACR 1A. Above, ^1^H NMR spectrum following incubation with active AMACR; below, ^1^H NMR spectrum following incubation with heat-inactivated AMACR. Red and green circles show methyl group signals for substrate **30** and unsaturated product **31**, respectively.

Incubation of a mixture of **30**/**31** with active AMACR confirmed that **30** was converted to **31**, as judged by reduction of the doublet at 0.96 ppm and appearance of the Me-group singlet at 1.97 ppm (Fig. 2). A change in the ratio of the triplets at 2.32 and 2.37 ppm (CH_2_ groups in the CoA side-chain) was also observed. These changes were not observed when using heat-inactivated enzyme, showing that the elimination was enzyme-catalysed. Product **31** absorbs at a maximum wavelength of <340 nm and this is not ideally suited for use in a microtitre plate assay. It was anticipated that addition of further electron-withdrawing groups or extension of the aromatic system would increase the wavelength of the product chromophore to >340 nm, but this would result in higher levels of HF elimination from the acid when conversion of the acyl-CoA ester was attempted. Therefore, further development of this approach was not undertaken.

**Fig. 3:**
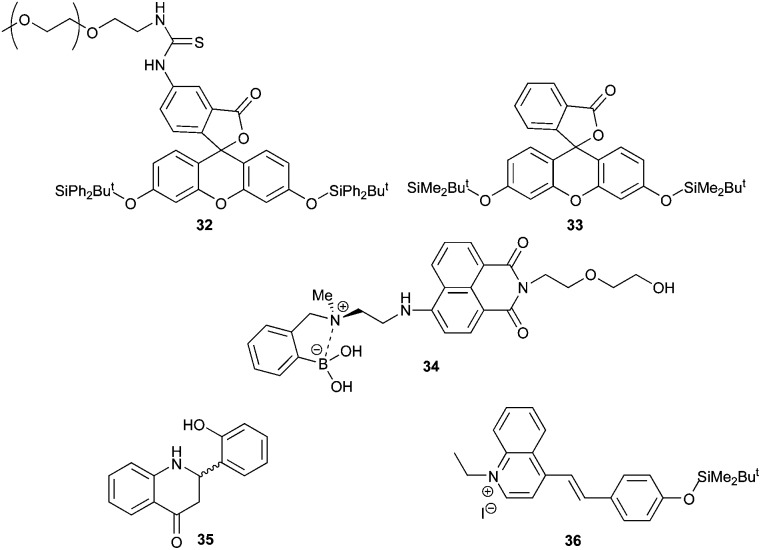
Fluoride sensors selected for study.

A second approach to developing a colorimetric or fluorescent assay for AMACR is to utilise a molecular fluoride sensor in order to measure the fluoride released during the enzymatic reaction. An advantage of this approach is that it allows assaying of a wide variety of potential AMACR substrates, including those with alkyl side-chains.^35^ Although there are many fluoride sensors reported, few of them can be used in aqueous systems. Fluorescent sensors **32**,^42^ **33**,^40^ **34**,^47^ and **35** ^43^ and the colorimetric sensor **36** ^41^ (Fig. 3) were selected for investigation due to their apparent sensitivity and compatibility with aqueous systems.

Synthesis of these selected sensors and incubation with fluoride in buffered aqueous solution was undertaken to validate the method. Sensor **32** initially gave a low fluorescent signal which rapidly increased with time, regardless of whether fluoride was present or not. This was also true with solutions prepared using highly purified water. It was concluded that spontaneous loss of the silyl-protecting group occurred due to the formation of the highly stabilised aromatic fluorescein. Sensor **33**, which has previously been used to assay γ-butyrobetaine hydroxylase activity,^40^ was therefore investigated. This sensor was more stable, but large variations in signal intensity at low aqueous fluoride concentrations were observed, limiting its use in enzymatic assays (ESI, Fig. S25†). Fluorescent detection of fluoride was also attempted using the ‘turn off’ sensor **34**. Reaction of strong nucleophiles such as fluoride^35^ results in fluorescent quenching of **34** due to a weakening of the interaction between the nitrogen and boron. However, incubation of **34** with standard fluoride solutions resulted in highly variable readings in aqueous solutions even in the presence of high organic solvent concentrations (ESI, Fig. S26 and S27†).

**Scheme 6:**
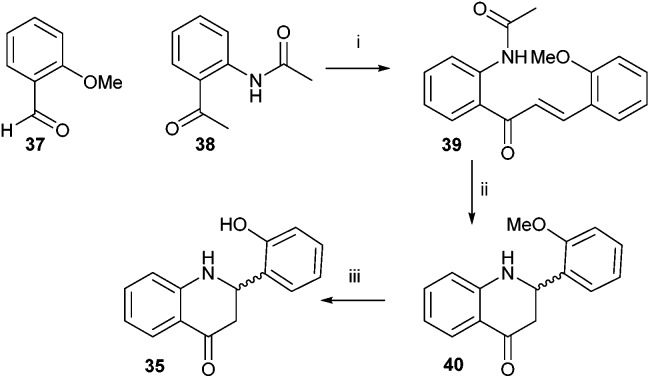
Synthesis of sensor **35**. Reagents and conditions: i. 5% (w/v) NaOH aq., MeOH, rt, 72%; ii. 5% (v/v) HCl aq., reflux, 62%; iii. NaSEt, DMF, 140 °C, 80%.

Sensor **35** was also investigated, as it is reported to be highly sensitive and to work by a mechanism that does not involve silyl-protecting group removal.^43^ Synthesis was accomplished by modification of the literature procedure (Scheme 6).^43^ 2-Hydroxybenzaldehyde was protected with MeI to give **37** and 2-aminoacetophenone was protected with acetyl chloride to give **38**. Compounds **37** and **38** were condensed together under alkali conditions to give **39**. Hydrolysis of the acetyl group from **39**, followed by cyclisation gave **40**. Removal of the O-methyl group with NaSEt gave **35**, whose structure was confirmed by X-ray crystallography (see ESI† for details). However, no fluorescence was observed for **35** in the presence of fluoride. It was noted that the spectroscopic data for **35** did not match that reported in the literature.^43^

**Scheme 7:**
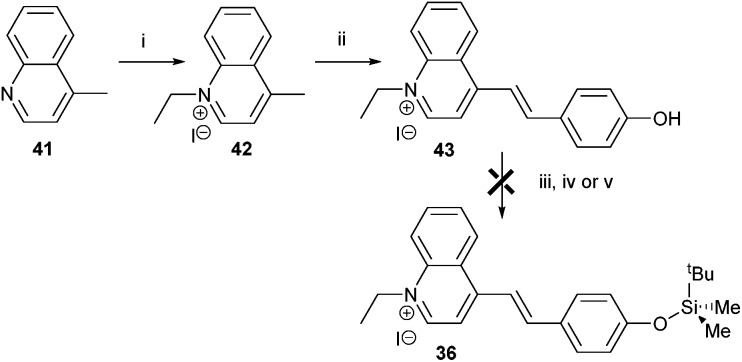
Attempted synthesis of fluoride sensor **36**. Reagents and conditions: i: ethyl iodide, toluene, reflux, 89%; ii: 4-hydroxybenzaldehyde, piperidine, MeOH, reflux, 60%; iii: TBDMSiCl, pyridine, reflux, 0%; iv: TBDMSiCl, NEt_3_, DCM, rt, 0%; v: TBDMSiCl, NEt_3_, CHCl_3_, reflux, 0%.

Finally, a colorimetric method for determining fluoride concentrations was investigated. The protected cyanine dye system **36** reported by Zhu *et al.*,^41^ was chosen since this is reported to be a highly sensitive system. The required dye was synthesised by reaction of lepidine **41** with ethyl iodide followed by coupling of the product **42** with 4-hydroxybenzaldehyde to give **43** (Scheme 7). However, protection of **43** with TBDMSiCl to give **36** could not be achieved using a number of different conditions, including those originally reported.^41^

## Conclusions

The results herein demonstrate that the elimination reaction catalysed by AMACR can be used to evaluate the potency of inhibitors. Conversion of substrate **1** to product **2** and HF was reduced in the presence of known AMACR substrates (acting as competitive inhibitors) and known inhibitors. *N*-Dodecyl-*N*-methyl-carbamoyl-CoA **8** gave the largest reduction in the conversion of **1** to **2** of all the acyl-CoA esters, consistent with previous reports^18^ that it is a potent inhibitor. Some of the inhibitors reported by Wilson *et al.* ^19^ also potently inhibited the enzyme. The throughput of this assay is not sufficient for high-throughput screening purposes, but it may allow preliminary characterisation of inhibitors identified through other approaches and would be a useful secondary screen for inhibitors identified using other methods.

Attempts to adapt the elimination assay to produce a colorimetric or fluorescent read-out were not very successful. Acyl-CoA substrates with aromatic side-chains were synthesised, but the presence of the aromatic side-chain resulted in loss of stereochemistry upon introduction of the fluoride leaving group. Loss of stereochemical integrity limits the application of these substrates as non-enzymatic elimination occurs in substrates in which the methyl group and fluorine atom are in a *syn*-arrangement. This approach is also limited by fluoride elimination upon conversion of the acid to the acyl-CoA. The results show that AMACR catalyses the elimination of fluoride from acyl-CoAs with aromatic side-chains and hence extends the range of known substrates.

The alternative approach of assaying AMACR activity by quantifying fluoride using sensors was also not very successful. Although there are several fluoride sensors reported for use in aqueous solutions, the high levels of hydration of the fluoride anion^35^ makes such methods slow and it can be difficult to achieve sufficient reproducibility.

## Experimental

### Sources of materials

All chemicals were obtained from the Sigma-Aldrich Chemical Co. or Fisher Scientific Ltd and were used without further purification, unless otherwise noted. Reagents were of analytical grade or equivalent (synthesis) or biochemical grade. Oasis HLB cartridges were obtained from Waters Corporation. Construction of the expression plasmid for human AMACR 1A has been previously described.^4^ The Rosetta2 (DE3) expression strain and auto-induction media system 1 were obtained from Novagen. (2*R*,3*R*)-3-Fluoro-2-methyldecanoyl-CoA **1** was synthesised as previously described.^35^ Fenoprofenoyl-CoA **3**, Flurbiprofenoyl-CoA **4**, Ibuprofenoyl-CoA **5**, Ketoprofenoyl-CoA **6** and Naproxenoyl-CoA **7** were synthesised as previously described.^5^ Ebselen **9** and Ebselen oxide **10** were obtained from Cayman Chemical. Compounds **14** ^48^ and **16** ^49^ were synthesised by their reported methods. The PEGylated fluorescein derivative **32** was synthesised by the method of Zheng *et al.* ^42^ *tert*-Butyldimethylsilyl-protected fluorescein **33** was synthesised by the method of Rydzik *et al.* ^40^ The boronic acid sensor **34** was synthesised by the method described by Sun *et al.* ^47^ Intermediates **37**,^50^ **38**,^51^ **39**,^52^ and **40** ^52^ for the synthesis of sensor **35** were produced by known methods. Intermediates **42** ^53^ and **43** ^53^ required for the synthesis of **36** were produced by known methods.

### General experimental procedures

Solvents were removed using Büchi rotary evaporators. Thin layer chromatography was performed on Merck silica aluminium plates 60 (F254) and UV light, potassium permanganate or phosphomolibdic acid were used for visualisation. Column chromatography was performed using Fisher silica gel (particle size 35–70 micron). Purifications of acyl-CoA esters were performed by solid phase extraction using Oasis HLB 6cc (200 mg) extraction cartridges. Phosphate buffer was prepared from monobasic and dibasic potassium phosphates at the required proportion for 0.1 M pH 7.0 buffer. Optical rotations were recorded on an Optical Activity AA-10 Automatic polarimeter instrument and are reported in 10^–1^ deg cm^2^ g^–1^. IR spectra were recorded on Perkin-Elmer RXI FTIR spectrometer instrument. NMR spectra were recorded on Bruker Avance III 400.04 MHz or 500.13 MHz spectrometers in D_2_O or CDCl_3_ and solvent was used as an internal standard. Shifts are given in ppm and *J* values reported to 0.1 Hz. Multiplicities are described as follows: s, singlet; d, doublet; t, triplet; q, quartet; m, multiplet. Stock concentrations of acyl-CoA esters for assays were determined using ^1^H NMR.^35^ Mass spectra were recorded by ESI TOF at the University of Bath Mass Spectrometry Service. High resolution mass spectra were recorded in ES mode. Aqueous solutions for biological experiments were prepared in 18.2 MΩ cm^–1^ Nanopure water and pH-adjusted with aq. HCl or NaOH. Syntheses were carried out at ambient temperature, unless otherwise specified. Solutions in organic solvents were dried over anhydrous magnesium sulfate and evaporated under reduced pressure.

### Synthesis of *N*-dodecyl-*N*-methyl-carbamoyl-CoA (**8**)^18^

Compound **8** was synthesised by the method of Carnell *et al.* ^18^ using 1-[dodecyl(methyl)carbamoyl]-3-methyl-1*H*-imidazol-3-ium iodide (40.0 mg, 0.092 mmol) and CoA-Li_3_ (17.0 mg, 0.020 mmol) in a mixture of dilute aqueous sodium hydrogen carbonate and THF. The crude aqueous product was freeze-dried and purified with solid phase extraction to obtain a white solid (7.1 mg). ^1^H NMR (500.13 MHz, CDCl_3_): *δ* 8.62 (1H, s), 8.35 (1H, s), 6.15 (1H, d, *J* = 6.0 Hz), 4.57–4.48 (1H, m), 4.23–4.13 (2H, m), 3.97 (1H, s), 3.84 (3H, s), 3.81–3.76 (1H, m), 3.53–3.47 (1H, m), 3.39 (2H, t, *J* = 6.5 Hz), 3.35–3.22 (3H, m), 2.95–2.82 (4H, m), 2.38 (2H, t, *J* = 6.5 Hz), 1.55–1.35 (2H, m), 1.25–1.05 (18H, m), 0.87 (3H, s), 0.76 (3H, t, *J* = 7.0 Hz), 0.72 (3H, s); HRMS (ES) [M + 2Na – 3H]^–^ Calcd. For C_35_H_60_N_8_Na_2_O_17_P_3_S: 1035.2805, found 1035.3050.

### Attempted synthesis of (*R*)-4-benzyl-3-[(2*S*,3*S*)-3-fluoro-2-methyl-3-phenylpropanoyl]oxazolidin-2-one (**15**)

A solution of DAST (0.09 mL, 0.68 mmol) in anhydrous DCM (1 mL) was added dropwise to a solution of **14** (230 mg, 0.68 mmol) in anhydrous DCM (3 mL) at –78 °C. The reaction mixture was stirred at –78 °C for 2 h, then allowed to reach ambient temperature. The reaction mixture was quenched by the slow addition of water (5 mL). The organic layer was washed with saturated NaHCO_3_ aq. and brine. The solution was dried over MgSO_4_, filtered and then the solvent was removed under reduced pressure. The product was purified by column chromatography (Pe : EtOAc 5 : 1) to give **15** as a mixture of diastereoisomers (200 mg, 87%, 50% de) as a colourless oil. ^1^H NMR (400.04 MHz, CDCl_3_): *δ* Major diastereomer (selected isolated peaks) 7.43–7.16 (10H, m), 5.63 (1H, dd, *J* = 46.4, 9.8 Hz), 4.80–4.72 (1H, m), 3.30 (1H, dd, *J* = 13.4, 3.4 Hz), 2.83 (1H, dd, *J* = 13.4, 9.5 Hz), 1.02 (3H, d, *J* = 7.0 Hz); minor diastereomer (selected isolated peaks) 7.43–7.16 (10H, m), 5.69 (1H, dd, *J* = 47.5, 6.6 Hz), 3.24 (1H, dd, *J* = 13.4, 3.3 Hz), 2.74 (^1^H, dd, *J* = 13.4, 9.7 Hz), 1.38 (3H, dd, *J* = 6.8, 0.8 Hz).

### Attempted synthesis of (2*S*,3*S*)-methyl-3-fluoro-2-methyl-3-phenylpropanoate (**17**)

A solution of DAST (92 μL, 0.70 mmol) in anhydrous DCM (1 mL) was added dropwise to a solution of **16** (135 mg, 0.70 mmol) in anhydrous DCM (4 mL) at –78 °C. The reaction mixture was stirred at –78 °C for 1 h, then allowed to reach ambient temperature. The reaction mixture was quenched by the slow addition of water (5 mL). The organic layer was washed with saturated NaHCO_3_ aq. and brine. The solution was dried over MgSO_4_, filtered and then the solvent was removed under reduced pressure. The product was purified by column chromatography (Pe : EtOAc 20 : 1) to give **17** (100 mg, 72%, 50% de) as a colourless oil. ^1^H NMR^54^ (400.04 MHz, CDCl_3_): *δ* Major diastereomer: 7.42–7.29 (5H, m), 5.54 (1H, dd, *J* = 46.1, 9.5 Hz), 3.77 (3H, s), 3.07–2.97 (1H, m), 0.96 (3H, d, *J* = 7.2 Hz); minor diastereomer: 7.42–7.29 (5H, m), 5.76 (1H, dd, *J* = 46.7, 6.1 Hz), 3.63 (3H, s), 2.97–2.87 (1H, m), 1.26 (3H, dd, *J* = 7.0, 0.8 Hz).

### Synthesis of (*S*)-4-benzyl-3-[(2*S*,3*S*)-3-hydroxy-2-methyl-3-(4-nitrophenyl)propanoyl]-oxazolidin-2-one (**20**)

A solution of dibutylboron triflate in DCM (1.0 M, 4.72 mL, 4.72 mmol) and diisopropylethylamine (0.90 mL, 5.14 mmol) were added to a stirred solution of oxazolidinone **19** (1.000 g, 4.29 mmol) in 10 mL of DCM at –78 °C and the resulting solution was stirred for 30 min at this temperature. *p*-Nitrobenzaldehyde **18** (0.907 g, 6.00 mmol) in 3 mL of DCM was added dropwise and the reaction mixture was stirred at –78 °C for 30 min and then allowed to reach ambient temperature. The reaction was quenched by slow addition of phosphate buffer (0.1 M, pH = 7.0, 10 mL), the organic layer was then washed with 1 M HCl aq., then saturated NaHCO_3_ aq. and brine. The solution was dried over MgSO_4_, filtered and then the solvent was removed under reduced pressure. The product was purified by column chromatography (Pe : EtOAc 2 : 1) to give **20** (1.460 g, 89%) as a yellow solid. m.p. 136–138 °C; [*α*]21D = +59.0 (CHCl_3_, *c* = 0.43); IR (KBr disc, cm^–1^): 3525.3, 1775.2, 1683.4 ^1^H NMR (400.04 MHz, CDCl_3_): *δ* 8.22 (2H, d, *J* = 8.7 Hz), 7.59 (2H, d, *J* = 8.7 Hz), 7.38–7.27 (3H, m), 7.23–7.17 (2H, m), 5.28–5.24 (1H, m), 4.78–4.70 (1H, m), 4.30–4.20 (2H, m), 4.05 (1H, dq, *J* = 7.0, 2.8 Hz), 3.54–3.48 (1H, m), 3.26 (1H, dd, *J* = 13.4, 3.4 Hz), 2.82 (1H, dd, *J* = 13.4, 9.4 Hz), 1.13 (3H, d, *J* = 7.0 Hz). ^13^C NMR (100.60 MHz, CDCl_3_) *δ* 176.67, 152.86, 148.34, 147.27, 134.66, 129.35, 129.01, 127.54, 126.93, 123.45, 72.19, 66.34, 55.01, 43.95, 37.71, 9.99. HRMS (ES) [M + H]^+^ Calcd. for C_20_H_21_N_2_O_6_: 385.1400, Found: 385.1421; [M + Na]^+^ Calcd. for C_20_H_20_N_2_NaO_6_: 407.1219, Found: 407.1216.

### Synthesis of (2*S*,3*S*)-methyl 3-hydroxy-2-methyl-3-(4-nitrophenyl)propanoate (**21**)

Compound **21** was synthesised by a new procedure: sodium metal (49 mg, 2.13 mmol) was reacted with anhydrous MeOH (20 mL), cooled to 0 °C, then treated with a solution of compound **20** (511 mg, 1.33 mmol) in anhydrous MeOH (5 mL). The resulting reaction mixture was stirred at 0 °C for 15 min. The reaction was quenched by the slow addition of phosphate buffer (0.1 M, pH = 7.0, 20 mL). The reaction mixture was extracted with DCM (4 × 20 mL) and the combined organic extracts were washed with brine, dried over MgSO_4_, filtered and the solvents were removed under reduced pressure. The product was purified by column chromatography (Pe : EtOAc 3 : 1) to give **21** (170 mg, 53%) as a yellow oil. ^1^H NMR^55^ (400.04 MHz, CDCl_3_): *δ* 8.23–8.17 (2H, m), 7.55–7.50 (2H, m), 5.24 (1H, m), 3.72 (3H, s), 3.32 (1H, d, *J* = 3.0 Hz), 2.79 (1H, dq, *J* = 7.3, 3.4 Hz), 1.07 (3H, d, *J* = 7.3 Hz).

### Synthesis of (2*R*,3*R*,*S*)-methyl-3-fluoro-2-methyl-3-(4-nitrophenyl)propanoate (**22**)

A solution of DAST (90 μL, 0.69 mmol) in anhydrous DCM (1 mL) was added dropwise to a solution of compound **21** (165 mg, 0.69 mmol) in anhydrous DCM (4 mL) at –78 °C. The reaction mixture was stirred at –78 °C for 1 h, then allowed to reach ambient temperature. The reaction mixture was quenched by the slow addition of water (5 mL). The organic layer was washed with saturated NaHCO_3_ aq. and brine. The solution was dried over MgSO_4_, filtered and then the solvent was removed under reduced pressure. The product was purified by column chromatography (Pe : EtOAc 20 : 1) to give **22** (93 mg, 56%, 77% de) as a colourless oil. ^1^H NMR (400.04 MHz, CDCl_3_): *δ* Major diastereomer: 8.27–8.19 (2H, m), 7.53–7.47 (2H, m), 5.71 (1H, dd, *J* = 45.8, 8.3 Hz), 3.75 (3H, s), 3.08–3.00 (1H, m), 1.02 (3H, d, *J* = 7.2 Hz); minor diastereomer: 8.27–8.19 (2H, m), 7.53–7.47 (2H, m), 5.87 (1H, dd, *J* = 46.5, 5.8 Hz), 3.66 (3H, s), 3.00–2.85 (1H, m), 1.25 (3H, dd, *J* = 7.1, 0.9 Hz).

### Synthesis of *syn*–*tert*-butyl 3-hydroxy-2-methyl-3-(4-nitrophenyl)propanoate (**24**)

*tert*-Butyl propionate **23** (1.00 mL, 865 mg, 6.64 mmol) was dissolved in anhydrous THF (20 mL), cooled to –78 °C, then lithium diisopropylamide in THF (2.0 M, 3.2 mL, 6.64 mmol) was added dropwise and the reaction mixture was stirred at this temperature for 30 min. 4-Nitrobenzaldehyde **18** (1004 mg, 6.64 mmol) in anhydrous THF (7 mL) was added to the reaction mixture, stirred for 2 h and then the reaction mixture was allowed to reach ambient temperature. The reaction mixture was quenched by slow addition of saturated NH_4_Cl aq. (20 mL), extracted with DCM. The organic layer was washed with water and brine, dried over MgSO_4_, filtered and the solvents were removed under reduced pressure. The residue was purified by column chromatography (Pe : EtOAc 10 : 1) to give compound **24** (970 mg, 52%) as a yellow oil. ^1^H NMR (400.04 MHz, CDCl_3_): *δ* 8.22–8.17 (2H, m), 7.55–7.50 (2H, m), 5.18 (1H, dd, *J* = 3.5, 2.8 Hz), 3.55 (1H, d, *J* = 2.8 Hz), 2.67 (1H, dq, *J* = 7.2, 3.5 Hz), 1.44 (9H, s), 1.03 (3H, d, *J* = 7.2 Hz). ^13^C NMR (100.59 MHz, CDCl_3_) *δ* 175.20, 148.80, 147.16, 126.87, 123.37, 81.81, 72.46, 46.27, 27.93, 10.33. HRMS (ES) [M + Na]^+^ Calcd. for C_14_H_19_NNaO_5_: 304.1161, Found: 304.1160.

### Synthesis of *anti*–*tert*-butyl 3-fluoro-2-methyl-3-(4-nitrophenyl)propanoate (**25**)

A solution of DAST (94 μL, 0.71 mmol) in anhydrous DCM (1 mL) was added dropwise to a solution of compound **24** (200 mg, 0.71 mmol) in anhydrous DCM (5 mL) at –78 °C. The reaction mixture was stirred at –78 °C for 2 h, then allowed to reach ambient temperature. The reaction mixture was quenched by the slow addition of water (10 mL). The organic layer was washed with saturated NaHCO_3_ aq. and brine. The solution was dried over MgSO_4_, filtered and then the solvent was removed under reduced pressure. The product was purified by column chromatography (Pe : EtOAc 30 : 1) to give **25** (102 mg, 51%) as white solid. ^1^H NMR (400.04 MHz, CDCl_3_): *δ* 8.26–8.21 (2H, m), 7.55–7.48 (2H, m), 5.78 (1H, dd, *J* = 46.8, 6.4 Hz), 2.89–2.74 (1H, m), 1.36 (9H, s), 1.23 (3H, dd, *J* = 7.1, 0.9 Hz). ^13^C NMR (125.76 MHz, CDCl_3_) *δ* 171.83 (d, *J* = 3.9 Hz), 148.15, 144.46 (d, *J* = 20.2 Hz), 127.35 (d, *J* = 7.0 Hz), 123.68, 94.25 (d, *J* = 176.6 Hz), 81.64, 46.99 (d, *J* = 24.6 Hz), 28.00, 12.92 (d, *J* = 6.7 Hz). ^19^F NMR (470.52 MHz, CDCl_3_) *δ* –175.39. HRMS (ES) [M + Na]^+^ Calcd. for C_14_H_18_FNNaO_4_: 306.1118, Found: 306.1106.

### Synthesis of *syn*-benzyl 3-hydroxy-2-methyl-3-(4-nitrophenyl)propanoate (**27**)

Benzyl propionate **26** (1.00 mL, 1.04 g, 6.33 mmol) was dissolved in anhydrous THF (20 mL), cooled to –78 °C, then LDA in THF (2.0 M, 3.2 mL, 6.33 mmol) was added dropwise and the reaction mixture was stirred at this temperature for 30 min. 4-Nitrobenzaldehyde **18** (956 mg, 6.33 mmol) in anhydrous THF (7 mL) was added to the reaction mixture, stirred for 2 h and then the reaction mixture was allowed to reach ambient temperature. Reaction mixture was quenched by slow addition of saturated NH_4_Cl aq. (20 mL) and extracted with DCM. The organic layer was washed with water and brine, dried over MgSO_4_, filtered and the solvents were removed under reduced pressure. The residue was purified by column chromatography (Pe : EtOAc 10 : 1) to give compound **27** (519 mg, 26%) as a yellow oil. ^1^H NMR^56^ (400.04 MHz, CDCl_3_): *δ* 8.19–8.12 (2H, m), 7.52–7.45 (2H, m), 7.38–7.26 (5H, m), 5.21 (1H, dd, *J* = 3.9, 3.2 Hz), 5.18–5.09 (2H, m), 3.21 (1H, d, *J* = 3.2 Hz), 2.84 (1H, dq, *J* = 7.2, 3.9 Hz), 1.12 (3H, d, *J* = 7.2 Hz). HRMS (ES) [M + Na]^+^ Calcd. for C_17_H_17_NNaO_5_: 338.1004, Found: 338.1006.

### Synthesis of *anti*-benzyl 3-fluoro-2-methyl-3-(4-nitrophenyl)propanoate (**28**)

A solution of DAST (0.46 mL, 3.45 mmol) in anhydrous DCM (10 mL) was added dropwise to a solution of compound **27** (989 mg, 3.14 mmol) in anhydrous DCM (20 mL) at –78 °C. The reaction mixture was stirred at –78 °C for 2 h, then allowed to reach ambient temperature. The reaction mixture was quenched by the slow addition of water (20 mL). The organic layer was washed with saturated NaHCO_3_ aq. and brine. The solution was dried over MgSO_4_, filtered and then the solvent was removed under reduced pressure. The product was purified by column chromatography (Pe : EtOAc 10 : 1) to give **28** (410 mg, 42%) as a colourless oil. ^1^H NMR (500.13 MHz, CDCl_3_): *δ* 8.23–8.16 (2H, m), 7.48–7.41 (2H, m), 7.40–7.31 (5H, m), 5.74 (1H, dd, *J* = 45.7, 7.9 Hz), 5.23–5.14 (2H, m), 3.14–3.03 (1H, m), 1.06 (3H, d, *J* = 7.2 Hz). ^13^C NMR (125.76 MHz, CDCl_3_) *δ* 172.25 (d, *J* = 4.2 Hz), 148.09, 143.91 (d, *J* = 20.4 Hz), 135.34, 128.58, 128.45, 128.31, 127.14 (d, *J* = 7.1 Hz), 123.65, 93.78 (d, *J* = 177.6 Hz), 66.88, 46.03 (d, *J* = 24.5 Hz), 12.66 (d, *J* = 6.3 Hz). ^19^F NMR (470.52 MHz, CDCl_3_) *δ* –176.09. HRMS (ES) [M + Na]^+^ Calcd. for C_17_H_16_FNNaO_4_: 340.0961, Found: 340.0947.

### Synthesis of *anti*-3-fluoro-2-methyl-3-(4-nitrophenyl)propanoic acid (**29**)

Compound **28** (216 mg, 0.68 mmol) was dissolved in anhydrous CHCl_3_ (10 mL), then TMSI (0.31 mL, 2.18 mmol) was added and the reaction mixture was stirred at 40 °C for 16 h. The reaction was quenched by slow addition of water (10 mL). The organic layer was washed with water and brine, dried over MgSO_4_, filtered and the solvent was removed under reduced pressure. The residue was purified by column chromatography (DCM : MeOH 10 : 1) to give **29** (139 mg, 90%) as a colourless oil. ^1^H NMR (500.13 MHz, CDCl_3_): *δ* 8.32–8.25 (2H, m), 7.58–7.51 (2H, m), 5.74 (1H, dd, *J* = 45.7, 8.6 Hz), 3.11–3.01 (1H, m), 1.08 (3H, d, *J* = 7.2 Hz). ^13^C NMR (125.76 MHz, CDCl_3_) *δ* 178.28, 148.32, 143.55 (d, *J* = 20.4 Hz), 127.40 (d, *J* = 6.9 Hz), 123.84, 93.68 (d, *J* = 177.1 Hz), 45.89 (d, *J* = 24.8 Hz), 12.81 (d, *J* = 6.5 Hz). ^19^F NMR (470.52 MHz, CDCl_3_) *δ* –173.92. HRMS (ES) [M + Na]^+^ Calcd. for C_10_H_10_FNNaO_4_: 250.0492, Found: 250.0477.

### Attempted synthesis of *anti*-3-fluoro-2-methyl-3-(4-nitrophenyl)propanoyl-CoA (**30**)

Compound **30** was prepared from the acid **29** using CDI and CoA-Li_3_ according to the usual procedure^5^ and purified with solid phase extraction to give a white powder. ^1^H NMR analysis showed that the product was a mixture of **30** and the eliminated acyl-CoA ester **31** in an approximate 4 : 1 ratio. Full characterisation was not possible, however selected peaks from the ^1^H spectrum of **30** can be reported. ^1^H NMR (500.13 MHz, D_2_O): *δ* 8.60 (1H, m), 8.34 (1H, m), 8.17 (2H, m), 7.53 (2H, m), 6.13 (1H, dd, *J* = 6.0, 2.0 Hz), 5.77–5.65 (2H, two overlapping dd, *J* = 45.5, 7.5 Hz),), 4.53 (1H, m), 4.20–4.14 (2H, m), 3.98 (1H, s), 3.82–3.75 (1H, m), 3.55–3.48 (1H, m), 3.42–3.22 (5H, m), 2.97 (2H, t, *J* = 6.8 Hz), 2.37 (2H, t, *J* = 6.8 Hz), 1.01 (3H, d, *J* = 7.2 Hz), 0.87 (3H, s), 0.74 (3H, s).

### Synthesis of 2-(2-hydroxyphenyl)-2,3-dihydroquinolin-4(1*H*)-one (**35**)^57^

NaSEt (2.602 g, 30.94 mmol) was added to a stirred solution of compound **40** (1.306 g, 5.16 mmol) in anhydrous DMF (46 mL) and the reaction mixture was stirred at 140 °C for 17 h. DMF was removed under reduced pressure, and the residue was dissolved in EtOAc, washed with saturated NH_4_Cl aq. and the organic layer was washed with water and brine, dried over MgSO_4_, filtered and the solvents were removed under reduced pressure. The residue was purified by column chromatography (Pe : EtOAc 5 : 1) to give compound **35** (990 mg, 80%) as a yellow solid. m.p. 179–181 °C, lit.^57^ 165–167 °C. IR (KBr disk, cm^–1^): 3096.5, 1639.1, 1607.6. ^1^H NMR (400.04 MHz, CDCl_3_): *δ* 7.94 (1H, dd, *J* = 7.9, 1.5 Hz), 7.66 (1H, s), 7.41 (1H, ddd, *J* = 8.2, 7.2, 1.6 Hz), 7.24 (1H, ddd, *J* = 8.2, 7.5, 1.7 Hz), 7.16 (1H, dd, *J* = 7.5, 1.6 Hz), 6.98–6.87 (3H, m), 6.83 (1H, d, *J* = 8.2 Hz), 4.89 (1H, ddd, *J* = 14.1, 3.6, 0.8 Hz), 4.70 (1H, br s), 3.10 (1H, dd, *J* = 16.8, 14.1 Hz), 2.83 (1H, ddd, *J* = 16.8, 3.6, 1.8 Hz). ^13^C NMR (125.77 MHz, CDCl_3_) *δ* 193.70, 155.21, 150.67, 135.51, 129.84, 127.90, 127.80, 124.86, 120.6, 120.46, 117.34, 117.25, 57.42, 43.62. HRMS (ES) [M + H]^+^ Calcd. for C_15_H_14_NO_2_: 240.1025, Found: 240.1005; [M + Na]^+^ Calcd. for C_15_H_13_NNaO_2_: 262.0844, Found: 262.0828. Details of the crystal structure determination and parameters are reported in the ESI.†

### Expression and purification of AMACR 1A

The plasmid for wild-type AMACR 1A^4^ was transformed into competent Rosetta2 (DE3) cells and plated onto Lennox LB media supplemented with 1% (w/v) agar, 30 μg mL^–1^ kanamycin sulfate and 32 μg mL^–1^ chloramphenicol. A single colony was picked into 10 mL Lennox LB media supplemented with 30 μg mL^–1^ kanamycin sulfate and 32 μg mL^–1^ chloramphenicol and grown overnight at 28 °C and 220 rpm. Growth of starter culture at 37 °C resulted in ‘leaky’ expression of wild-type AMACR. Starter culture was used to inoculate 500 mL of LB media supplemented with the same antibiotics and 1× auto-induction media and grown under the same conditions overnight. Cells were harvested (Beckman JA-10 rotor, 9000 rpm, 16 000*g*, 30 min) and stored at –80 °C.

Cells (∼2 g) were re-suspended in 30 mL start buffer and AMACR was purified as previously described.^35^ Fractions containing AMACR were identified by SDS-PAGE analyses using 10% gels, pooled and dialysed into 10 mM NaH_2_PO_4_–NaOH, pH 7.4. Protein concentrations were determined by absorbance at 280 nm, and extinction coefficients and molecular weights for the His-tag protein calculated using Protparam (; http://web.expasy.org/protparam/).

### ^1^H NMR assay of AMACR activity

Enzyme assays with inhibitor were performed using a similar method to previously reported.^35^ Enzyme (0.12 mg mL^–1^; 2.54 μM) was incubated with inhibitor (200 μM) in the presence of NaH_2_PO_4_–NaOH, pH 7.4 and *ca.* 88% (v/v) D_2_O (275 μL) for 10 min. An equal volume of (2*R*,3*R*)-3-fluoro-2-methyldecanoyl-CoA substrate **1** (200 μM) in buffer and D_2_O (275 μL) was added to the enzyme/inhibitor mixture, and the assay was incubated at 30 °C for 60 min. Enzyme was inactivated by heating at 60 °C for 10 min before ^1^H NMR analysis. Conversion of substrates was quantified by conversion of the 2-Me doublet at *ca.* 1.0 ppm into a singlet at *ca.* 1.75 ppm, and levels were corrected for non-enzymatic conversion in heat-inactivated negative controls (<5% conversion).^4,5^ Reported conversions are relative to positive controls lacking an inhibitor (100% activity). Approximately 50% of substrate **1** was converted to **2** after 1 h. Substrate conversion levels in the presence and absence of each inhibitor are given in the ESI (Table S1†) Concentrations of acyl-CoA substrate and inhibitor stock solutions were determined by ^1^H NMR.^5^

### Fluorescent detection of fluoride

Sensor **33** in DMSO (256 μM, 80 μL) was incubated with NaF (0–640 μM, 20 μL) in 50 mM Tris-HCl, pH 7.5 in a black microtitre plate at ambient temperature. After 1 h, 50 μL of 50 mM HEPES–NaOH, pH 7.0 was added. Fluorescence was determined using a FLUOstar Omega plate reader (BMG Labtech) with excitation wavelength 480 nm and emission wavelength 520 nm.^40^ The graph is shown in the ESI as Fig. S25.†

Sensor **34** (final concentration 2 μM) was incubated with fluoride (final concentrations 0–300 μM) in a total volume of 200 μL for 3 min. Reactions were carried out in 100% acetonitrile (using tetra-*n*-butylammonium fluoride) or in 50 mM NaH_2_PO_4_–NaOH, pH 7.4 (using NaF) and acetonitrile [1 : 19 (v/v)]. Fluorescence was determined using a FLUOstar Omega plate reader (BMG Labtech) with excitation wavelength 350 nm and emission wavelength 520 nm.^47^ The graphs are shown in the ESI as Fig. S26 and S27,† respectively.

## Abbreviations

AMACR: α-Methylacyl-CoA racemase (P504S)
CoA: Coenzyme A
CDI: Carbonyldiimidazole
DAST: Diethylaminosulfur trifluoride
DCM: Dichloromethane
de: Diastereomeric excess
DMF: Dimethylformamide
DMSO: Dimethylsulfoxide
D_2_O: Deuterium oxide
EtOAc: Ethyl acetate
EtOH: Ethanol
ESI TOF: Electrospray ionisation time-of-flight
HEPES: 4-(2-Hydroxyethyl)-1-piperazineethane sulfonic acid
HPLC: High performance liquid chromatography
HRMS: High resolution mass spectrometry
IR: Infra-red
LB: Luria-Bertani
MeI: Methyl iodide
MeOH: Methanol
m.p.: Melting point
NaSEt: Sodium ethanethiolate
NMR: Nuclear magnetic resonance
Pe: Petroleum ether
rpm: Revolutions per minute
ppm: Parts per million
SDS-PAGE: Sodium dodecyl sulfate polyacrylamide gel electrophoresis
TBDPSi-: *tert*-Butyldiphenylsilyl-
THF: Tetrahydrofuran
TMSI: Trimethylsilyl iodide
Tris: Tris(hydroxymethyl)methylamine
