# Supplementary material for: A study on the AMACR catalysed elimination reaction and its application to inhibitor testing
Source: Org Biomol Chem. 2015 Nov 5;14(2):612–22. doi: 10.1039/c5ob01541c (PMC4718014; doi:10.1039/c5ob01541c)
Supplement: Supplementary file 1 [file OB-014-C5OB01541C-s001.pdf]

Electronic Supplementary Material (ESI) for Organic & Biomolecular Chemistry.
This journal is © The Royal Society of Chemistry 2015

Supplementary Information

A study on the AMACR catalysed elimination reaction and its application to inhibitor testing

Maksims Yevglevskis,[a] Guat L. Lee,[a] Jenny Sun,[a,b] Shiyi Zhao,[a,b] Xiaolong Sun,[c] Gabriele Kociok-Köhn,[c] Tony D. James,[c] Timothy J. Woodman,[a] and Matthew D. Lloyd[a]

[a]*Medicinal Chemistry, Department of Pharmacy & Pharmacology, University of Bath, Claverton Down, Bath BA2 7AY, United Kingdom.*

[b]*Department of Pharmacy, Shandong University, People's Republic of China.*

[c]*Department of Chemistry, University of Bath, Claverton Down, Bath BA2 7AY, United Kingdom.*

Abbreviations used: CoA, coenzyme A; DMSO, dimethylsulfoxide; HEPES, 4-(2-hydroxyethyl)-1-piperazineethane sulfonic acid; Tris, Tris(hydroxymethyl)methylamine

Contents

^{1}H, ^{13}C and ^{19}F NMR spectra………3

Single Crystal X-ray Crystallographic Data……………………………………………………………………………………..26

Fluorescence Experimental Procedures…………………………………………………………………………………………31

Figure S1. ^{1}H NMR spectrum of N-Dodecyl-N-methyl-carbamoyl-CoA (**8**)

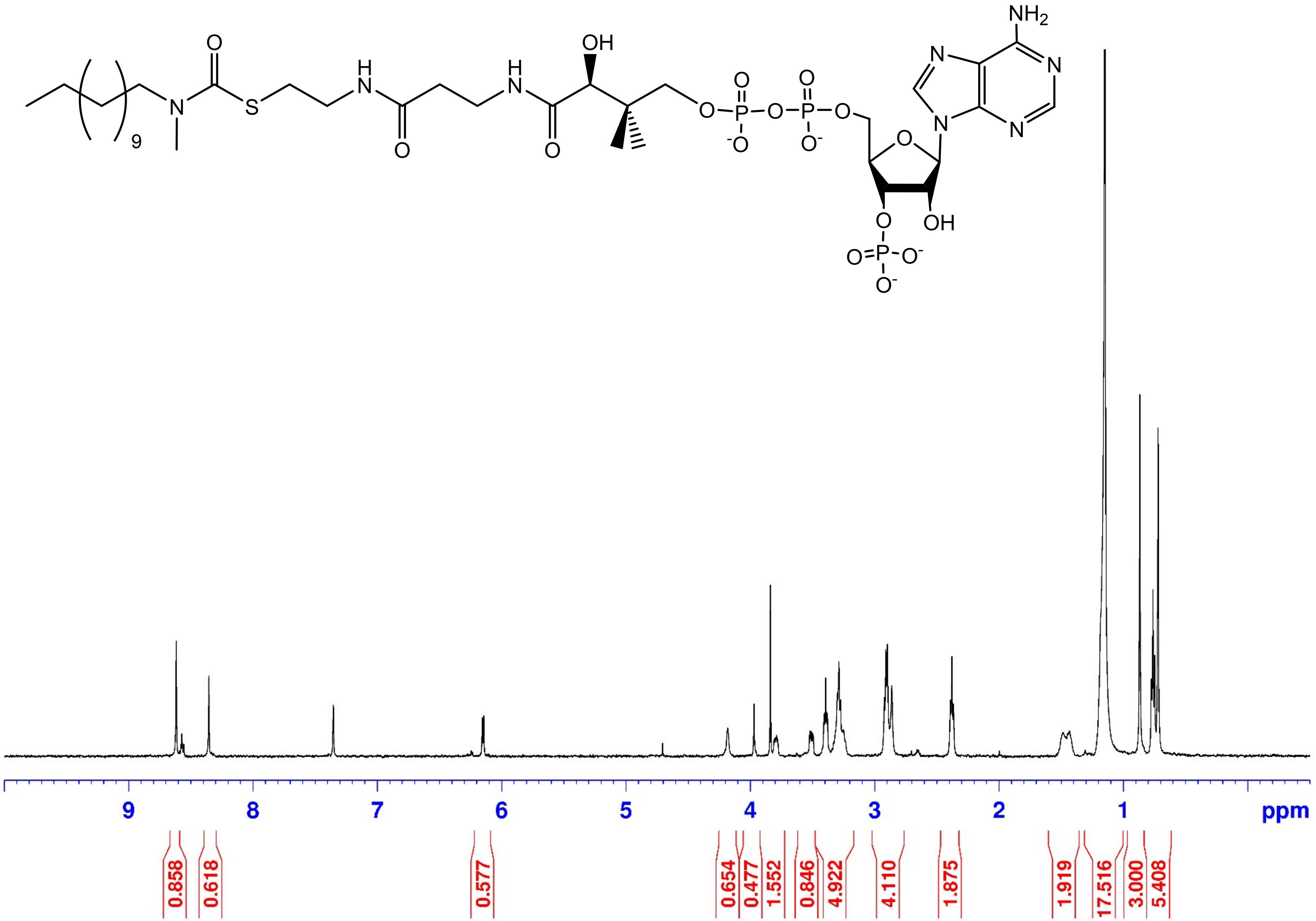

Figure S2. ^{1}H NMR spectrum of (*R*)-4-Benzyl-3-[(2*S*,3*S*)-3-fluoro-2-methyl-3-phenylpropanoyl]oxazolidin-2-one (**15**) mixture of diastereoisomers

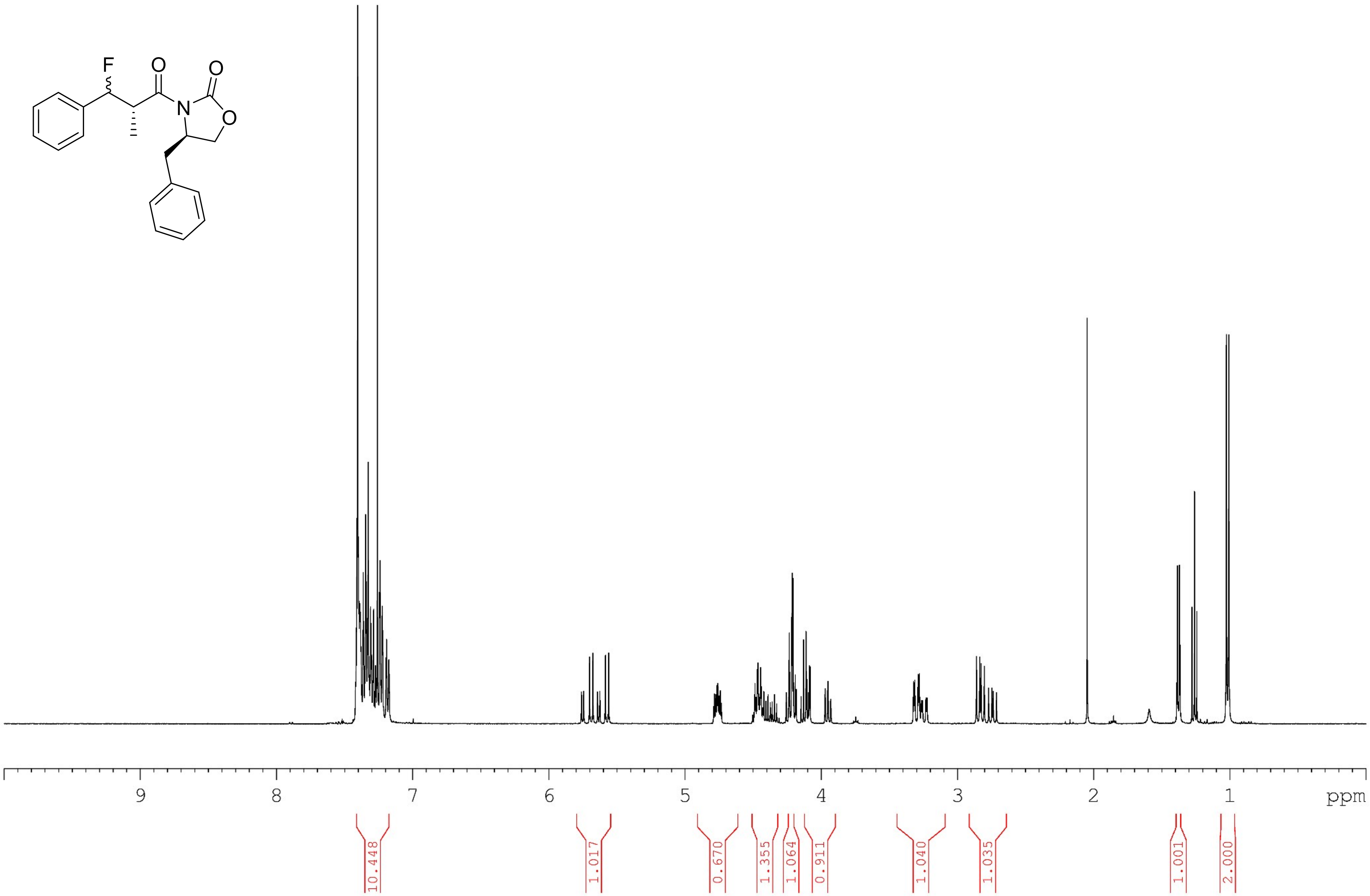

Figure S3. ^{1}H NMR spectrum of (2*S*,3*S*)-Methyl-3-fluoro-2-methyl-3-phenylpropanoate (**17**) mixture of diastereoisomers

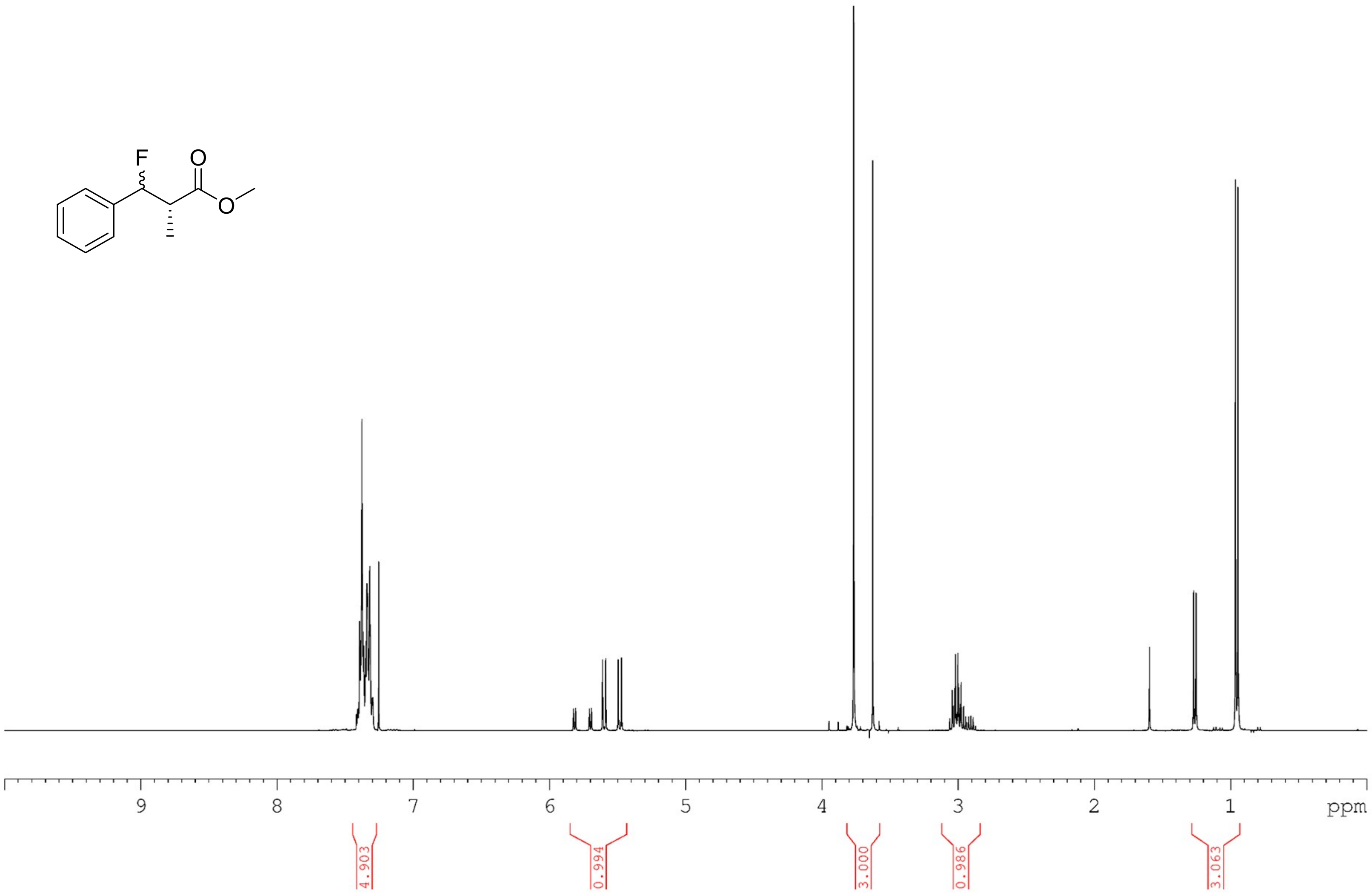

Figure S4. ^{1}H NMR spectrum of (*S*)-4-Benzyl-3-[(2*S*,3*S*)-3-hydroxy-2-methyl-3-(4-nitrophenyl)propanoyl]-oxazolidin-2-one (**20**)

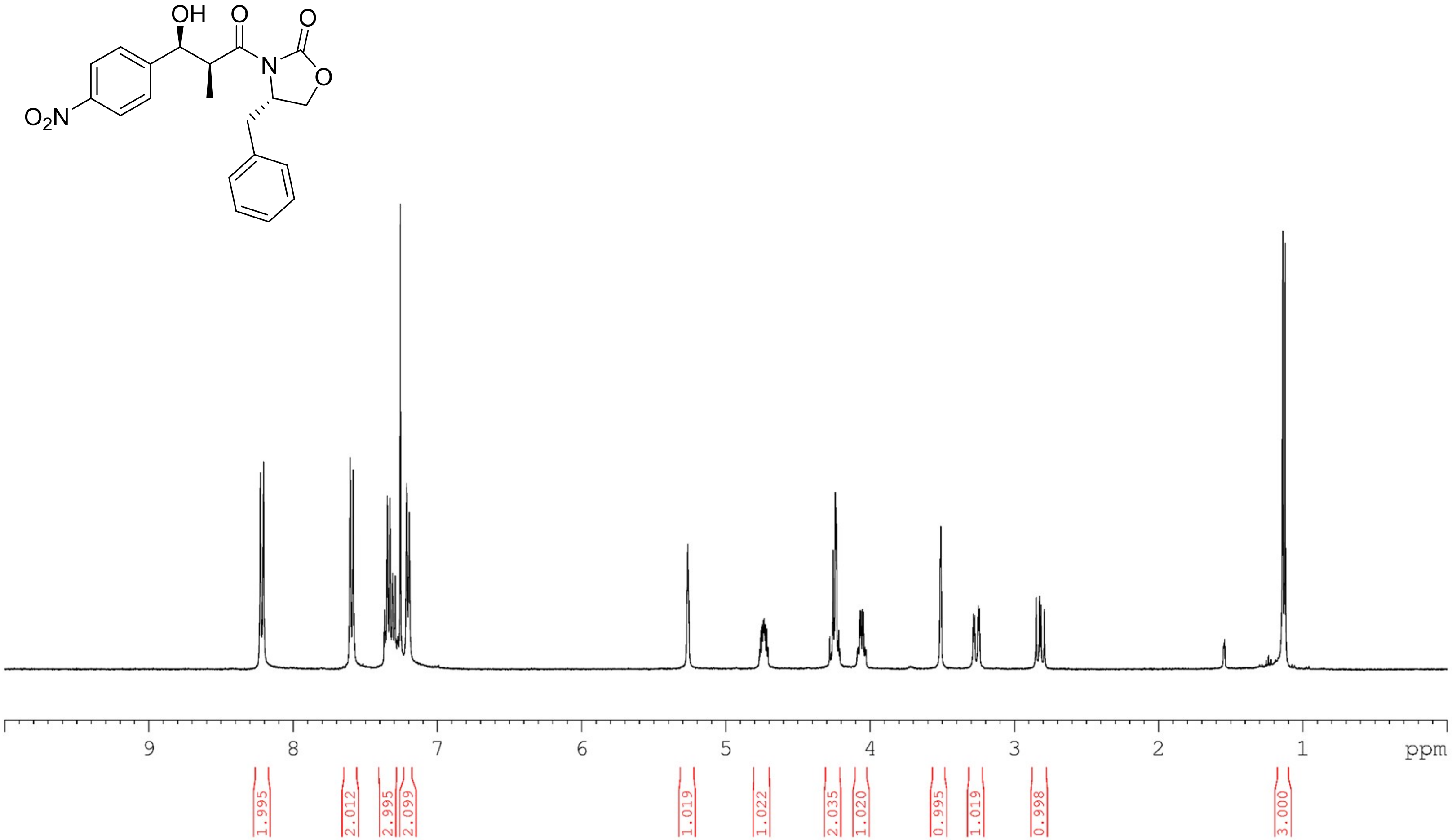

Figure S5. ^{13}C NMR spectrum of (*S*)-4-Benzyl-3-[(2*S*,3*S*)-3-hydroxy-2-methyl-3-(4-nitrophenyl)propanoyl]-oxazolidin-2-one (**20**)

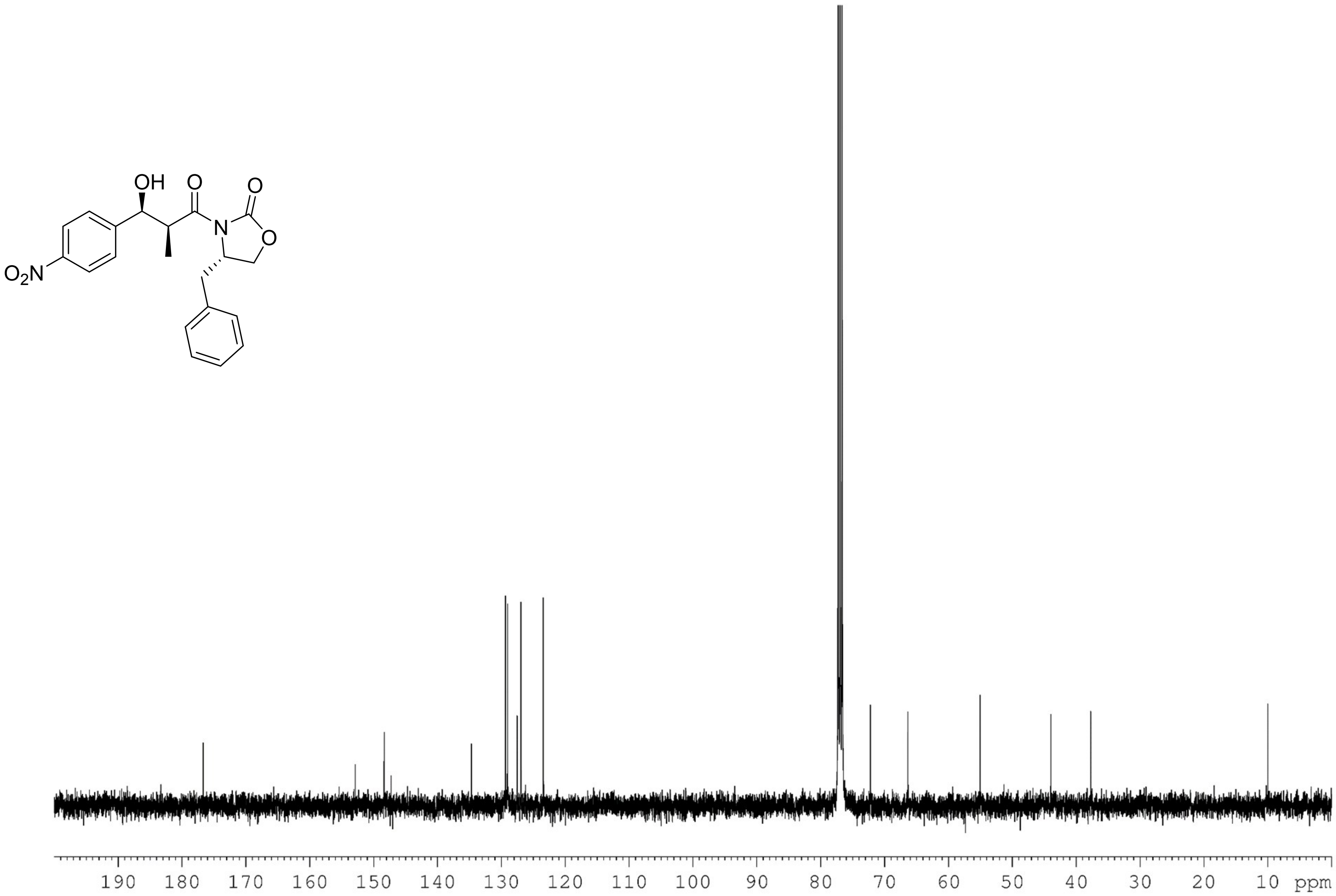

Figure S6. ^{1}H NMR spectrum of (2*S*,3*S*)-Methyl 3-hydroxy-2-methyl-3-(4-nitrophenyl)propanoate (**21**)

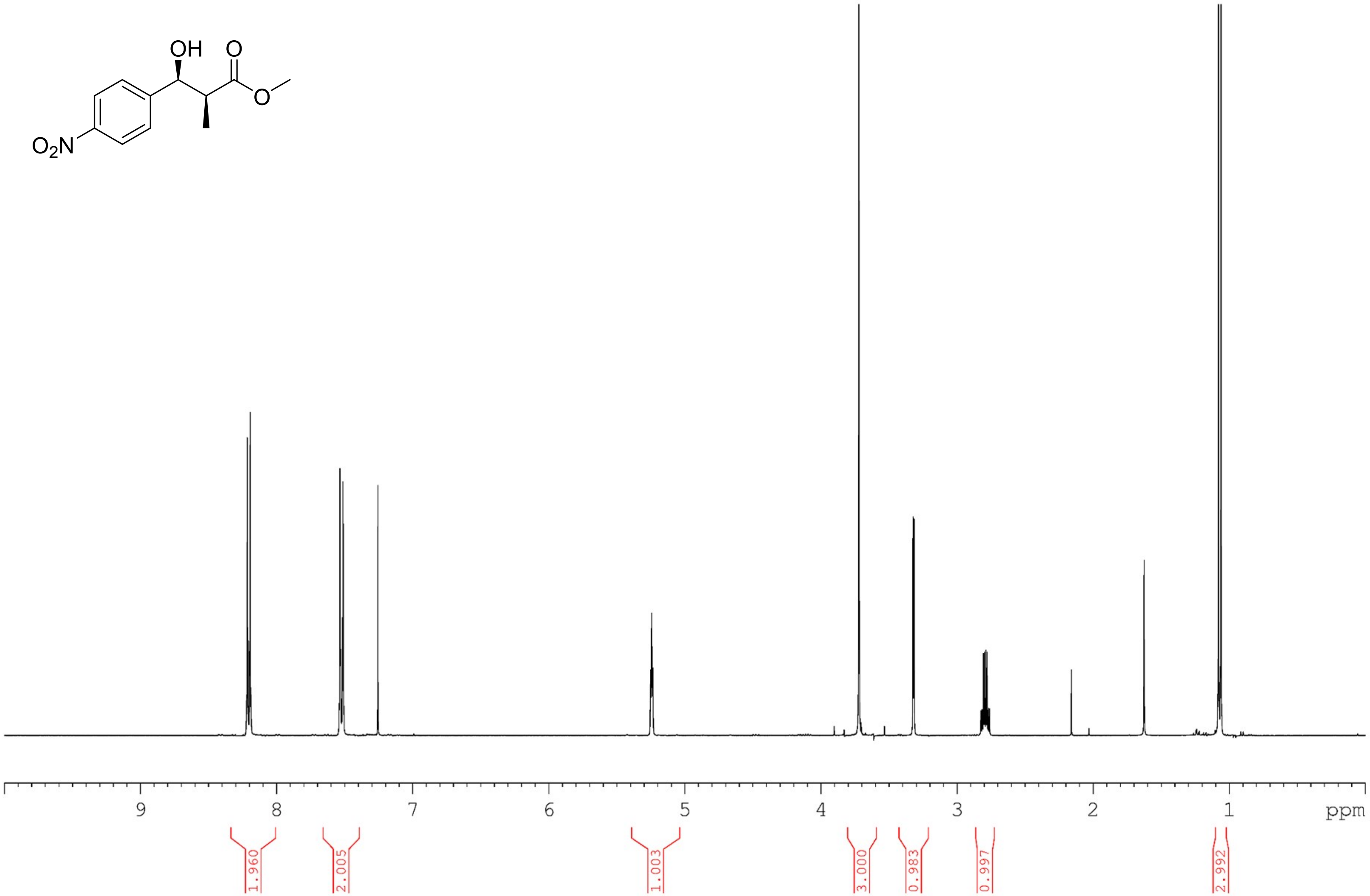

Figure S7. ¹H NMR spectrum of (2*R*,3*S*)-Methyl-3-fluoro-2-methyl-3-(4-nitrophenyl)propanoate (**22**) mixture of diastereoisomers

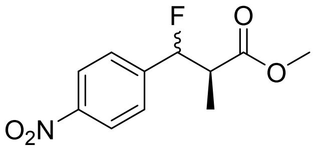

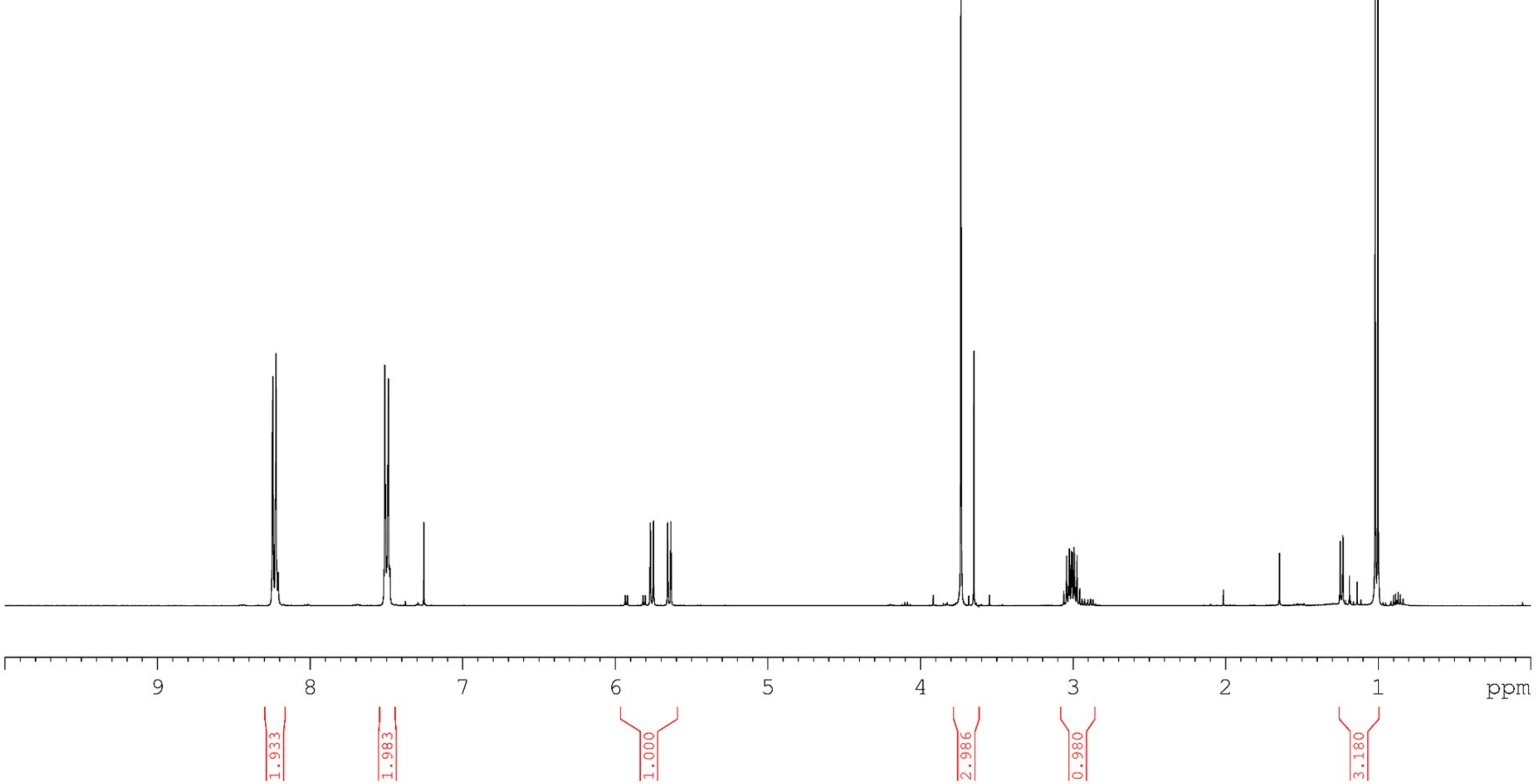

Figure S8. ¹H NMR spectrum of *syn-tert*-Butyl 3-hydroxy-2-methyl-3-(4-nitrophenyl)propanoate (**24**)

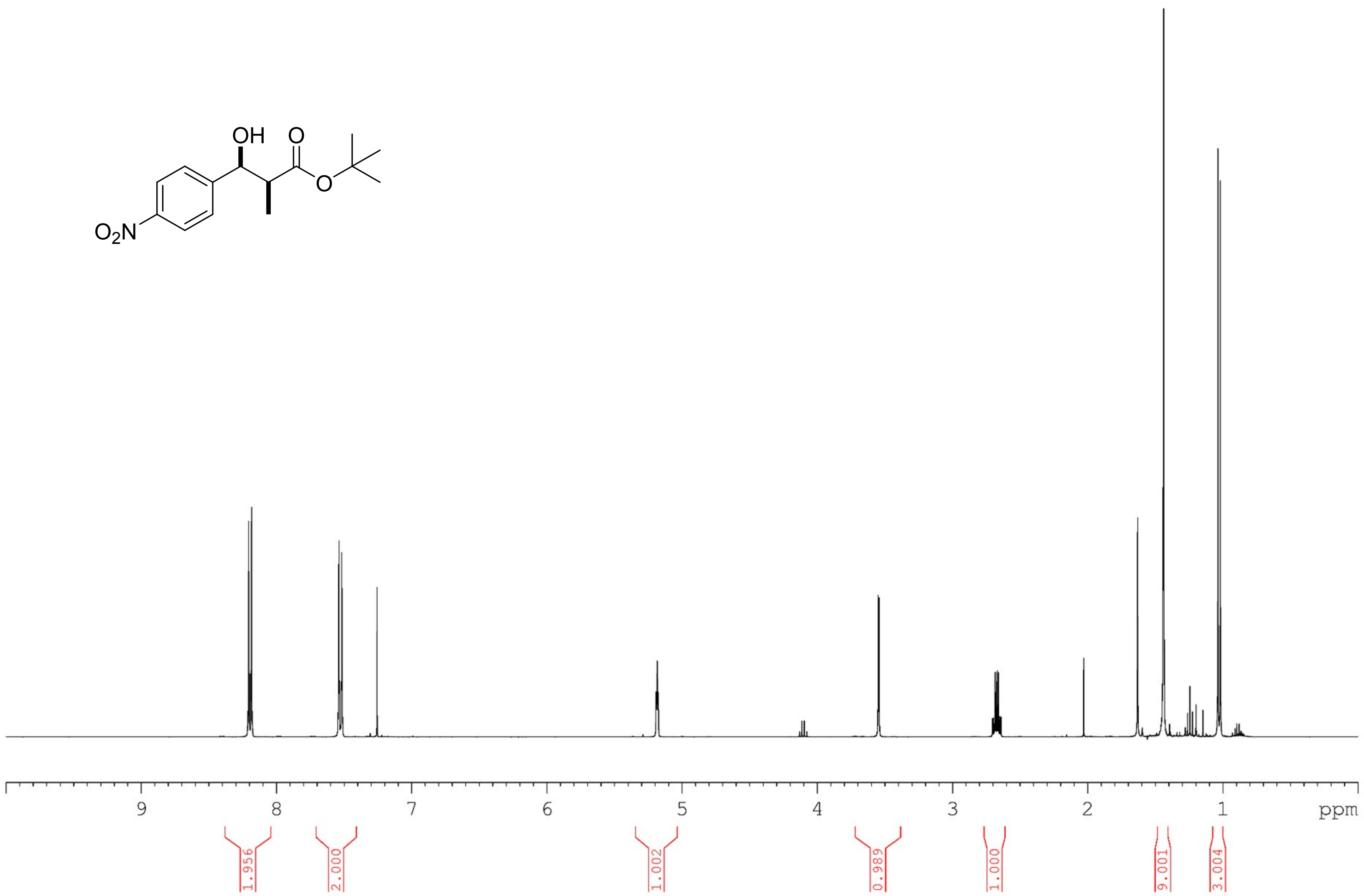

Figure S9. ^{13}C NMR spectrum of *syn-tert*-Butyl 3-hydroxy-2-methyl-3-(4-nitrophenyl)propanoate (**24**)

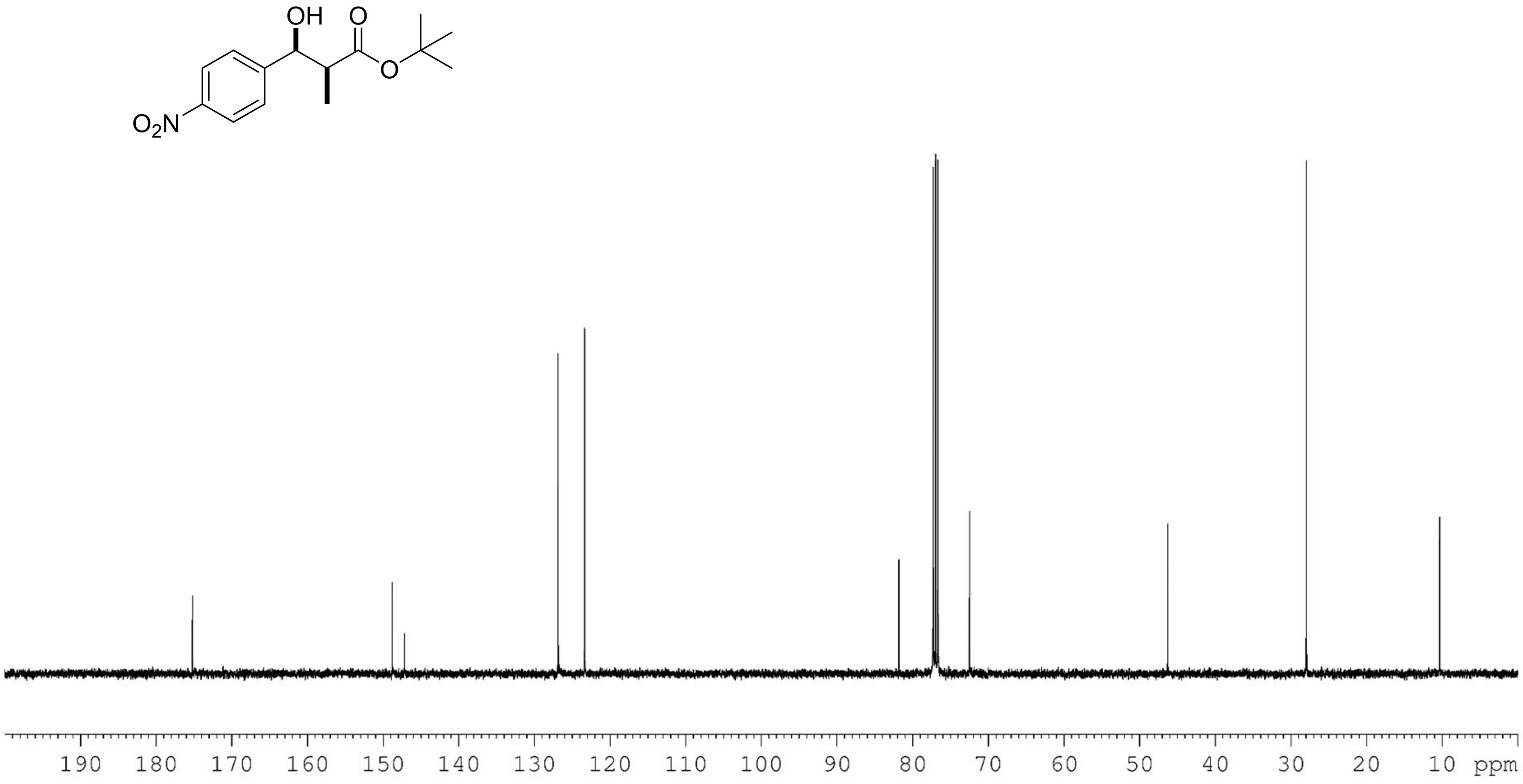

Figure S10. ^{1}H NMR spectrum of *anti-tert*-Butyl 3-fluoro-2-methyl-3-(4-nitrophenyl)propanoate (**25**)

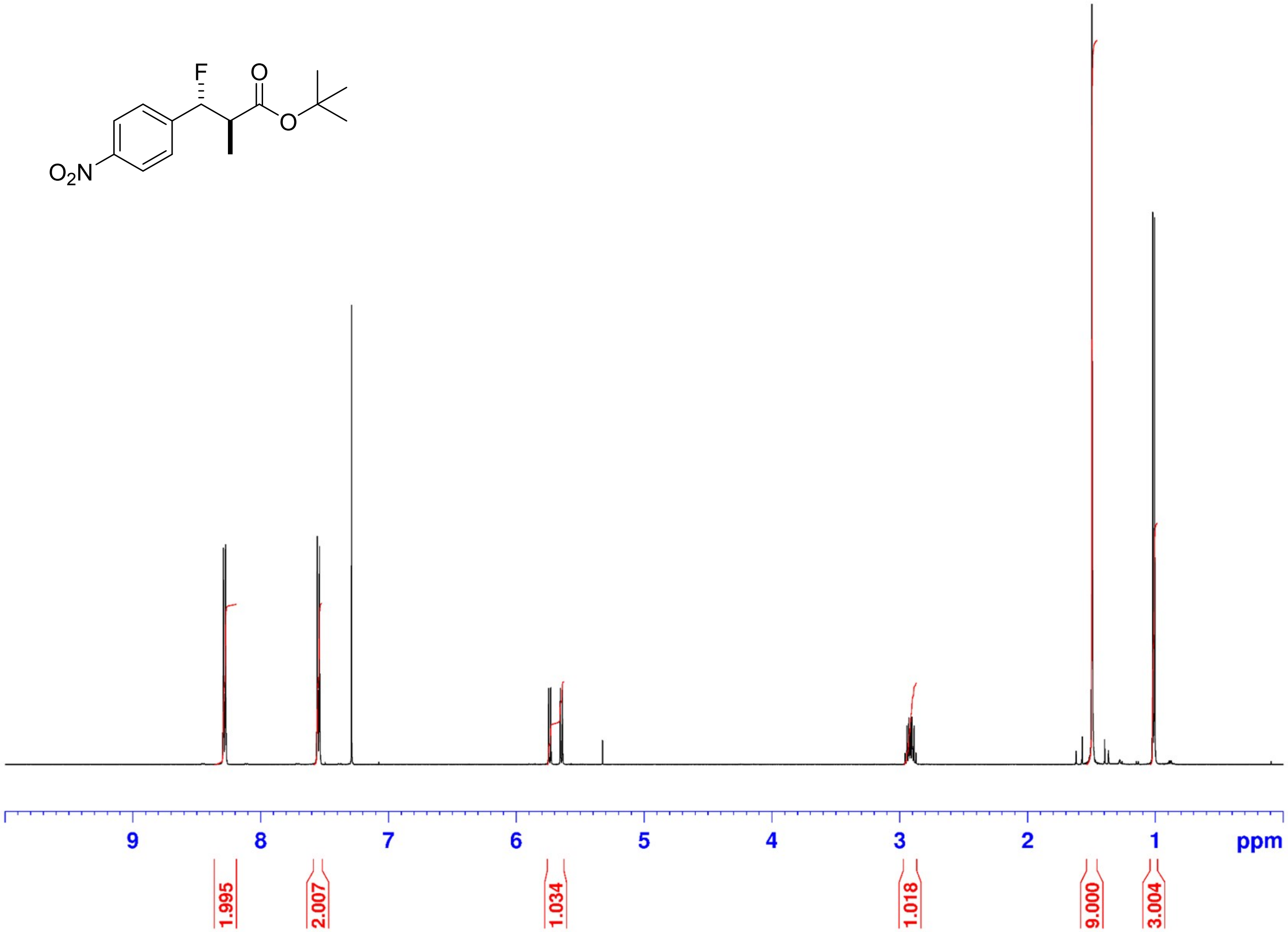

Figure S11. ^{13}C NMR spectrum of *anti*-*tert*-Butyl 3-fluoro-2-methyl-3-(4-nitrophenyl)propanoate (**25**)

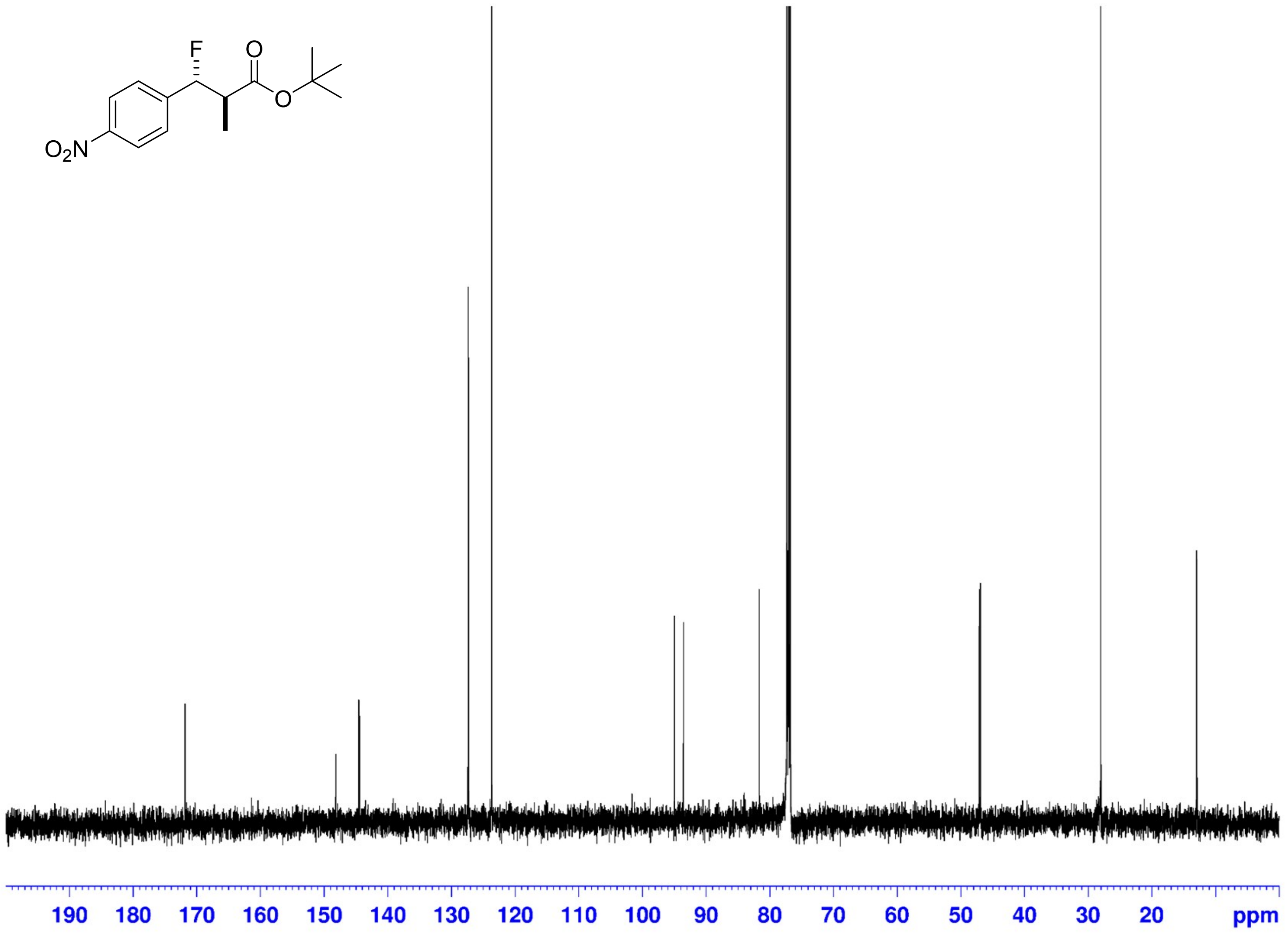

Figure S12. ^{19}F NMR spectrum of *anti*-*tert*-Butyl 3-fluoro-2-methyl-3-(4-nitrophenyl)propanoate (**25**)

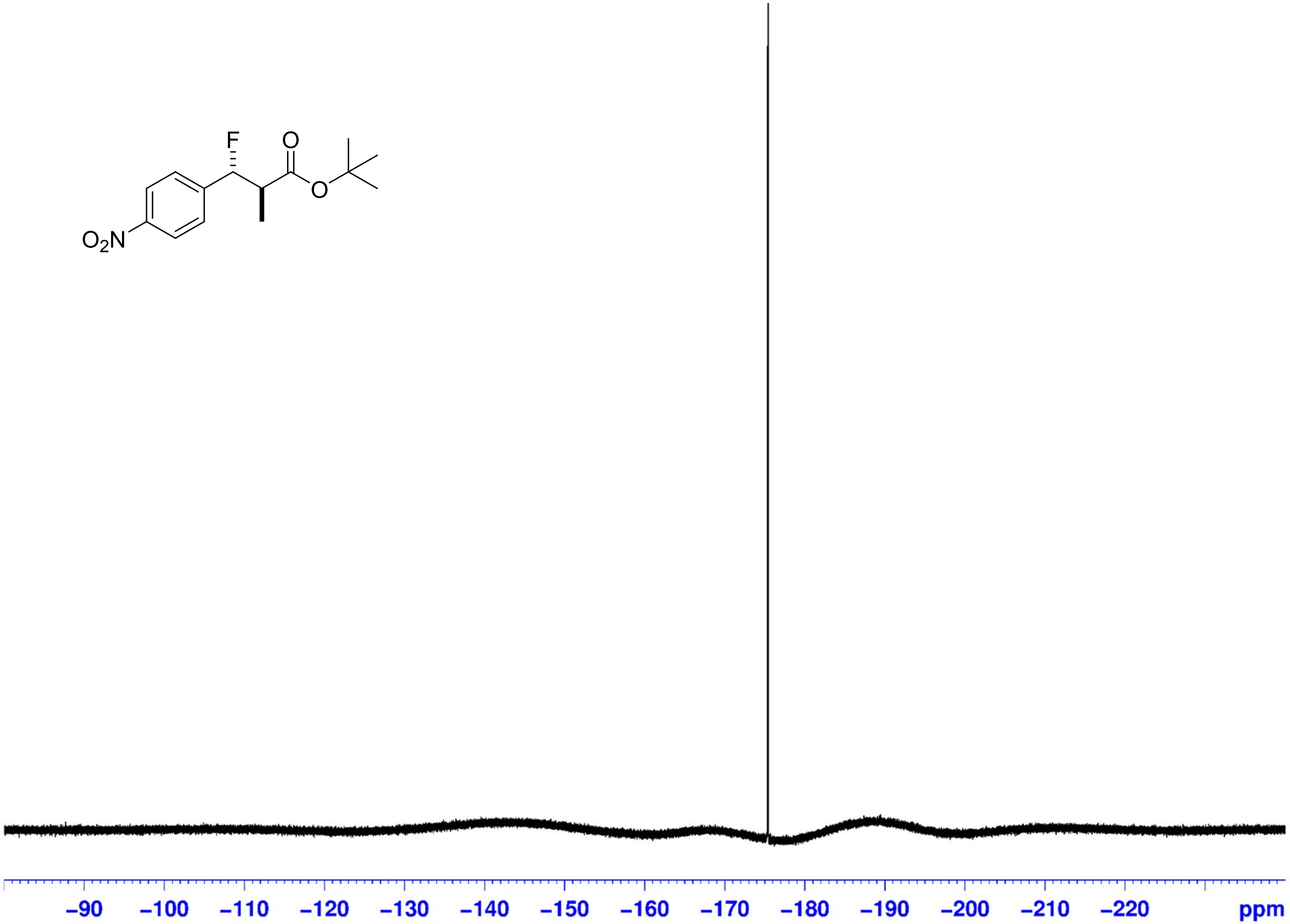

Figure S13. ^{1}H NMR spectrum of *syn*-Benzyl 3-hydroxy-2-methyl-3-(4-nitrophenyl)propanoate (**27**)

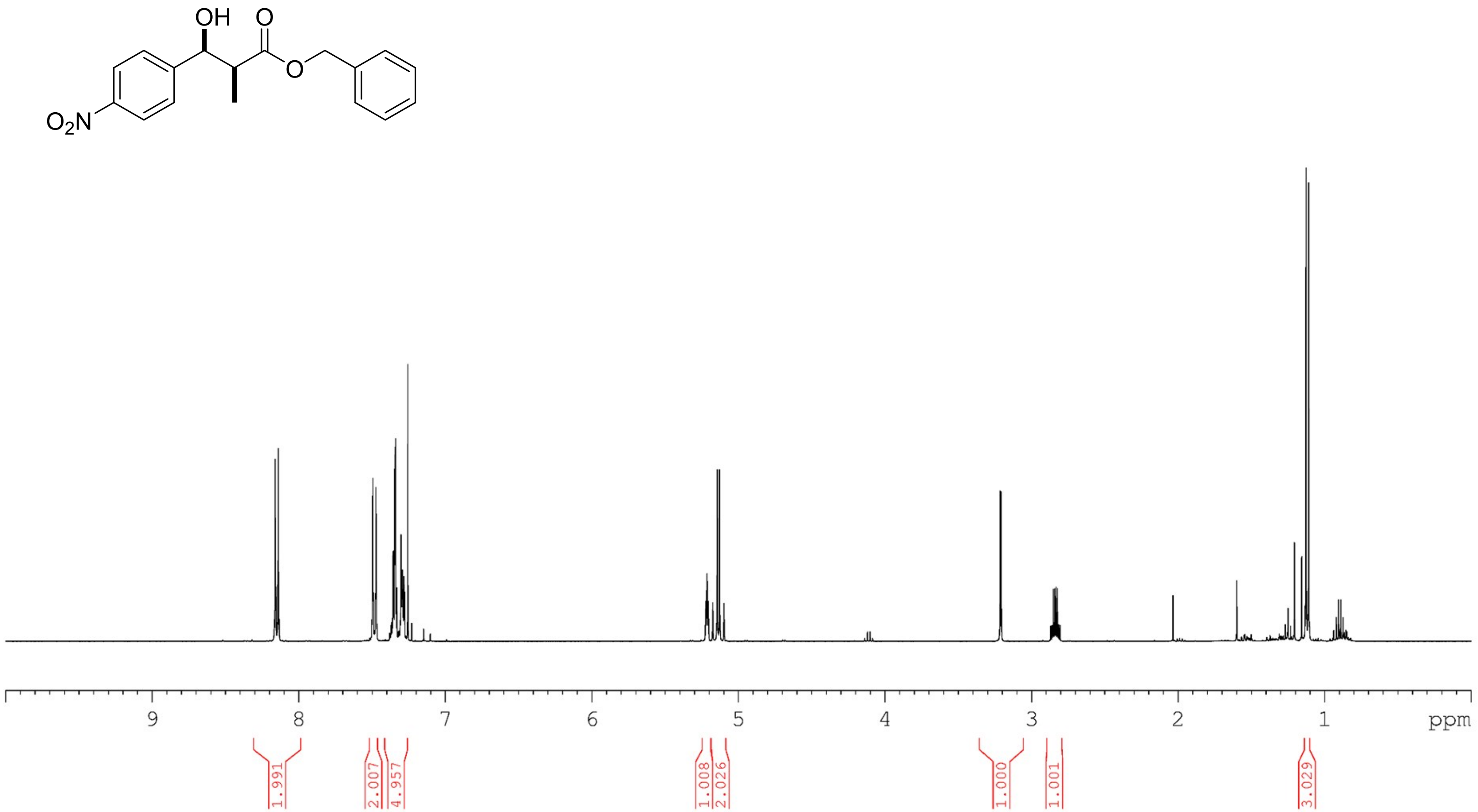

Figure S14. ^{1}H NMR spectrum of *anti*-Benzyl 3-fluoro-2-methyl-3-(4-nitrophenyl)propanoate (**28**)

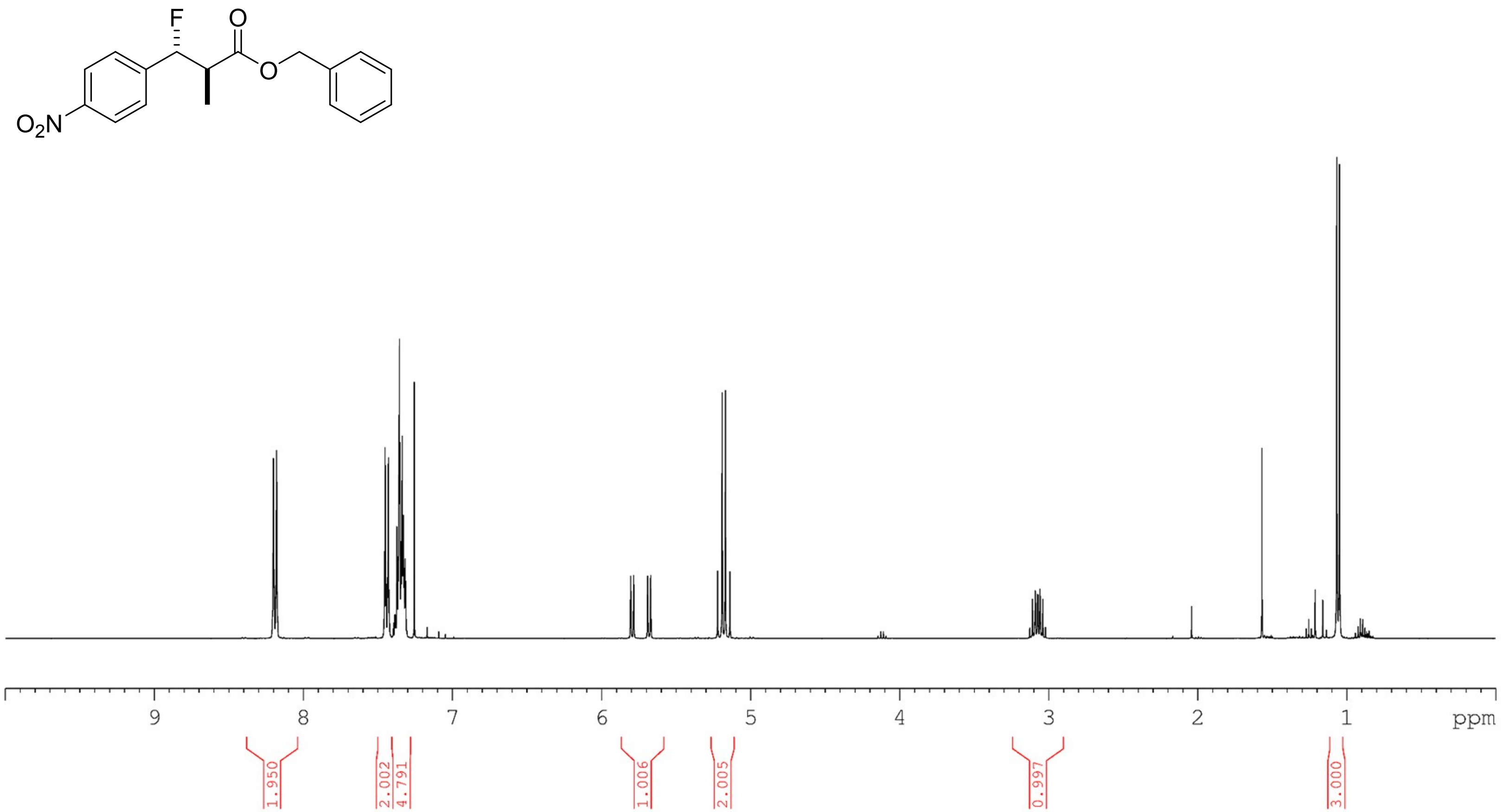

Figure S15. ^{13}C NMR spectrum of *anti*-Benzyl 3-fluoro-2-methyl-3-(4-nitrophenyl)propanoate (**28**)

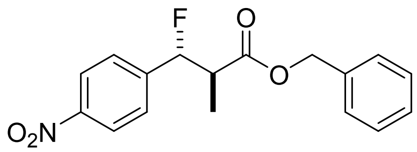

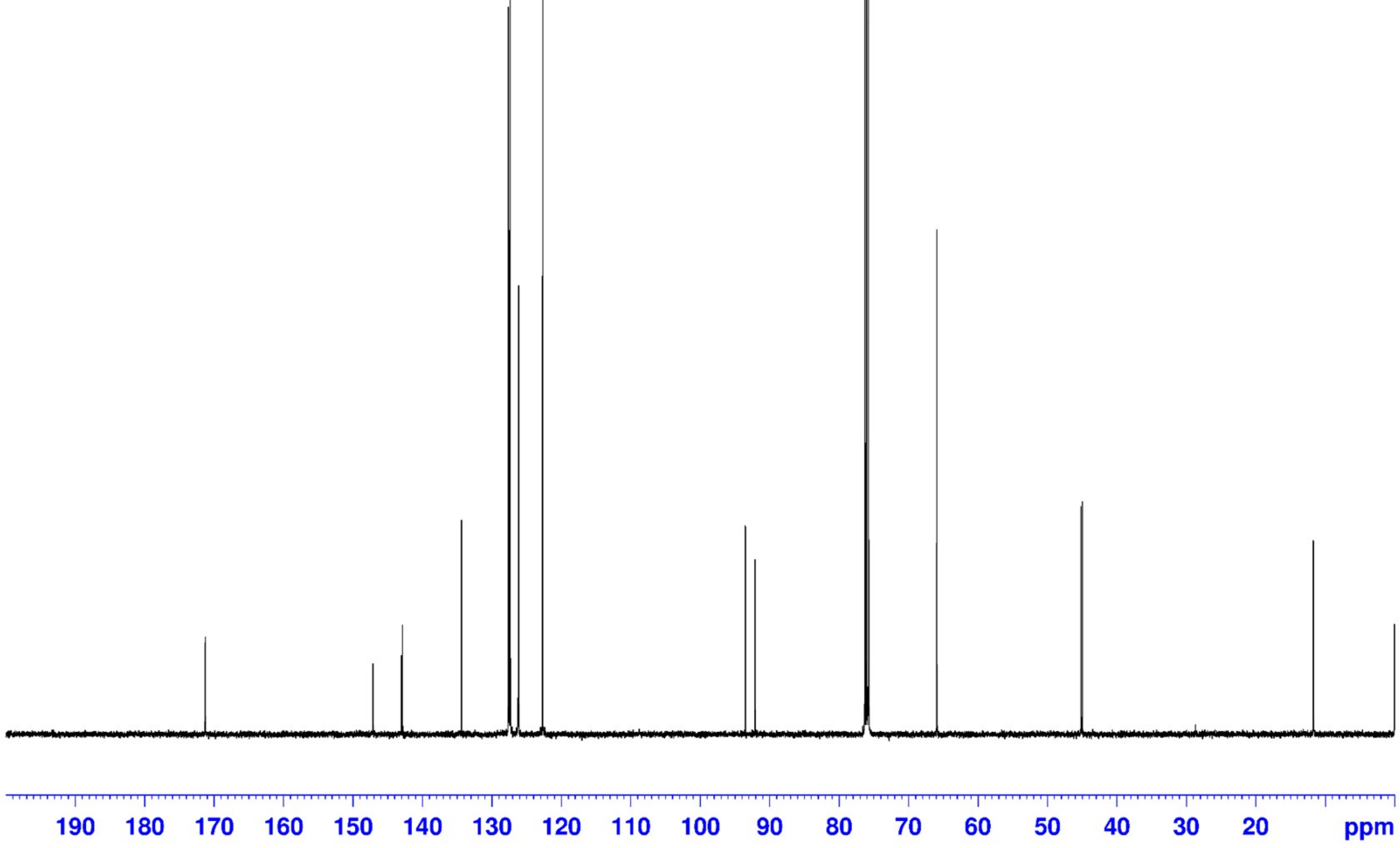

Figure S16. ^{19}F NMR spectrum of *anti*-Benzyl 3-fluoro-2-methyl-3-(4-nitrophenyl)propanoate (**28**)

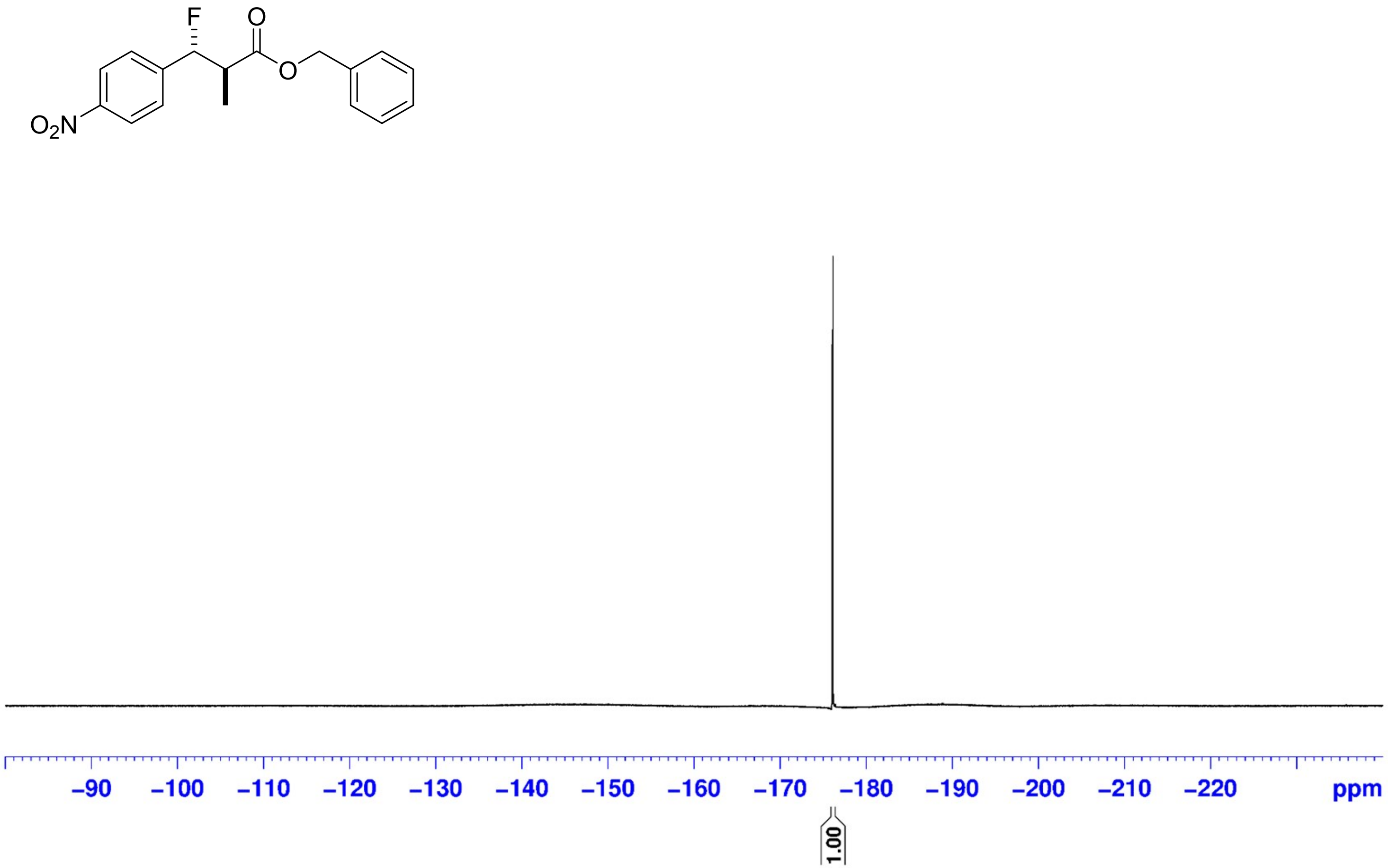

Figure S17. ^{1}H NMR spectrum of *anti*-3-Fluoro-2-methyl-3-(4-nitrophenyl)propanoic acid (**29**)

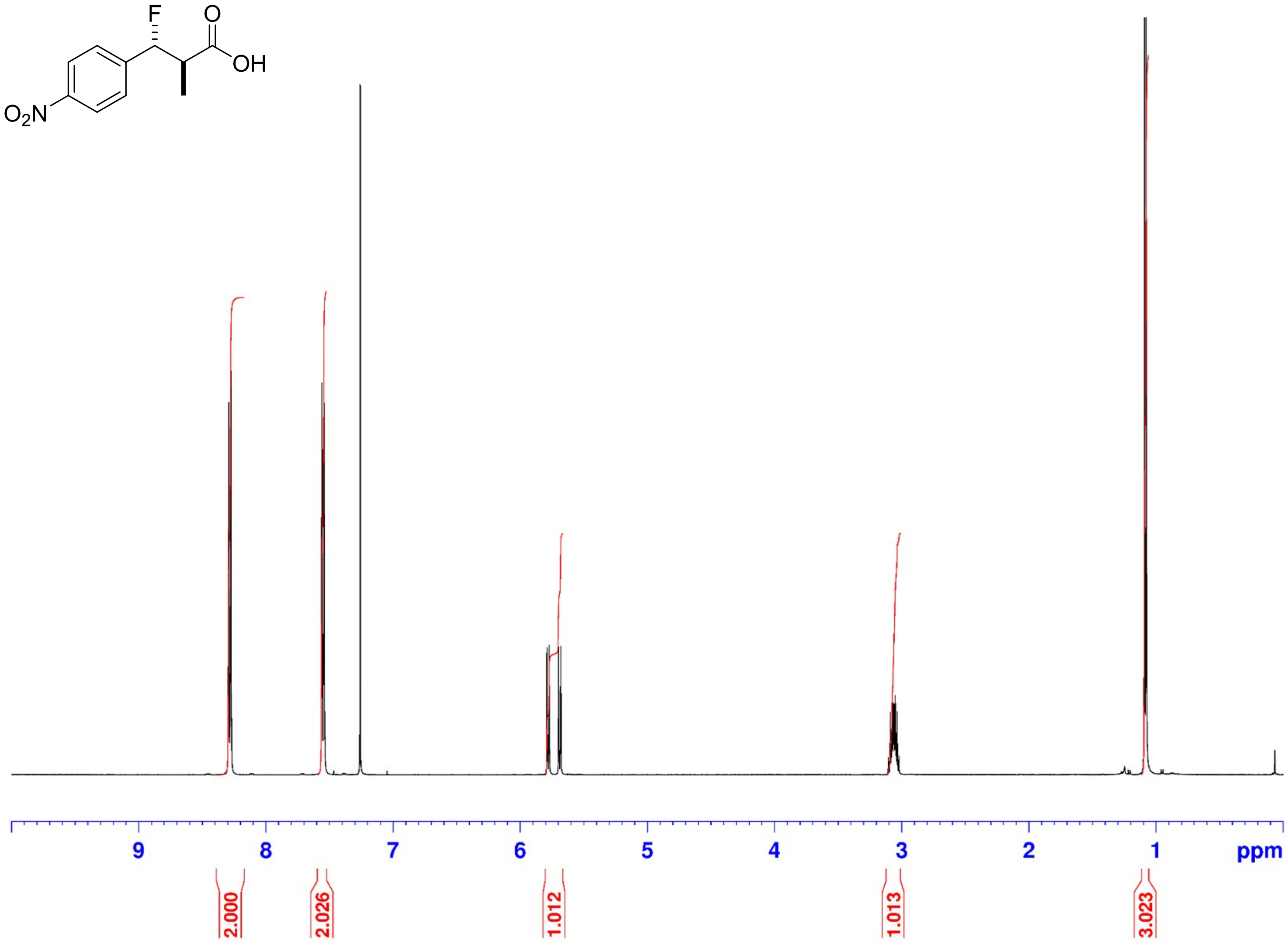

Figure S18. ^{13}C NMR spectrum of *anti*-3-Fluoro-2-methyl-3-(4-nitrophenyl)propanoic acid (**29**)

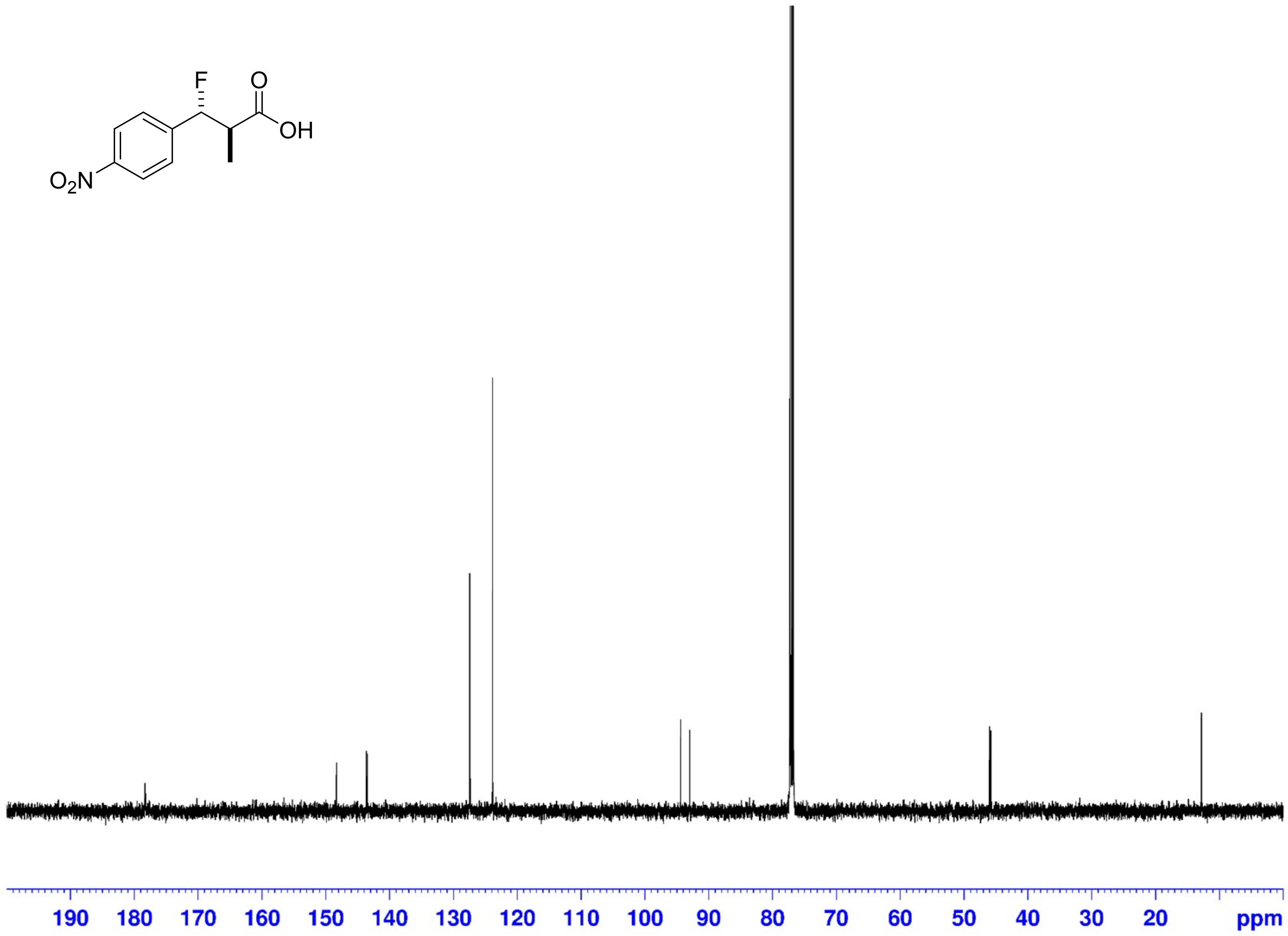

Figure S19. ^{19}F NMR spectrum of *anti*-3-Fluoro-2-methyl-3-(4-nitrophenyl)propanoic acid (**29**)

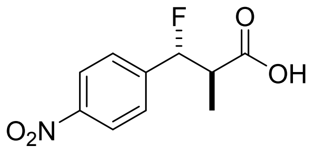

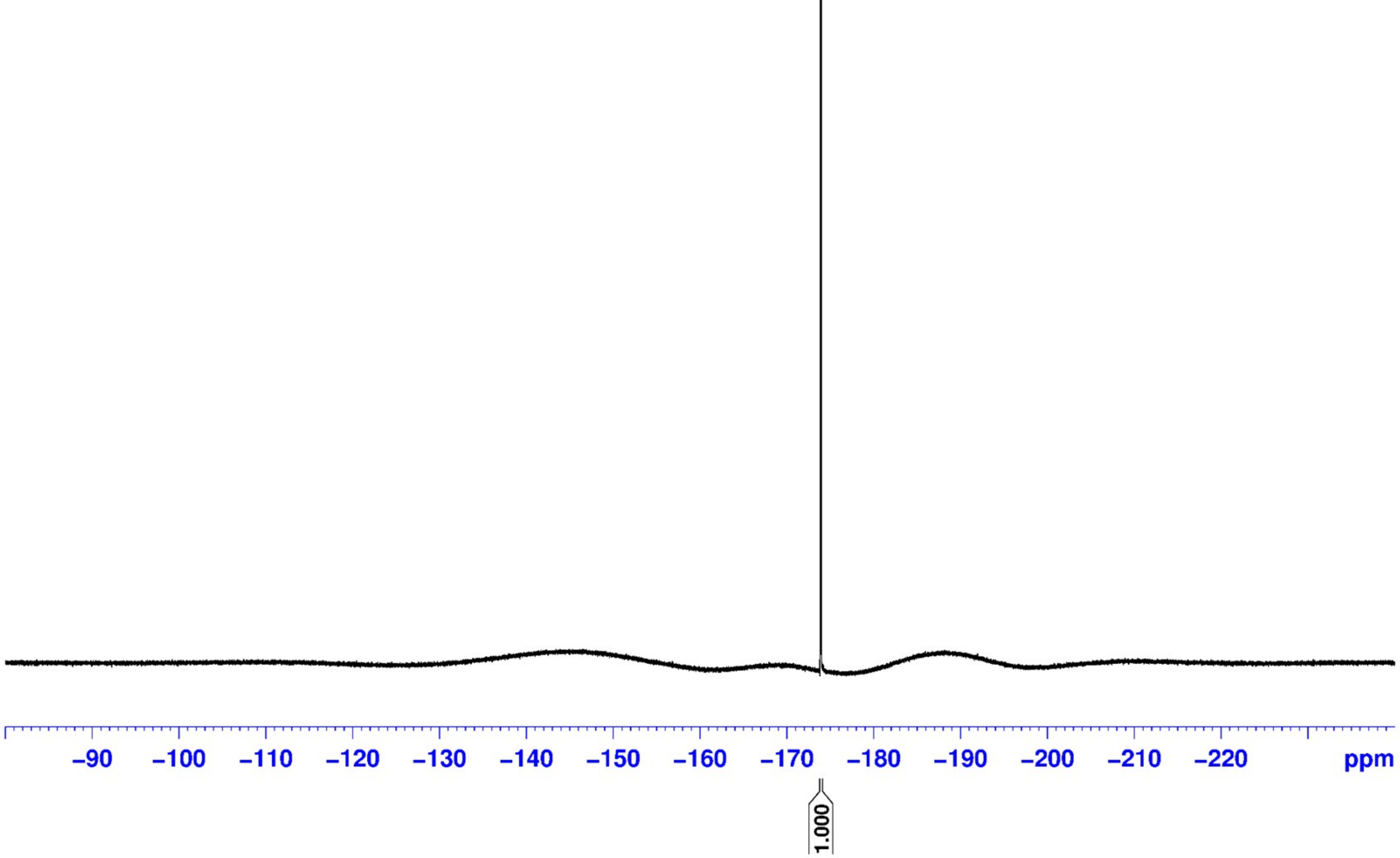

Figure S20. ^{1}H NMR spectrum *anti*-3-Fluoro-2-methyl-3-(4-nitrophenyl)propanoyl-CoA (**30**)

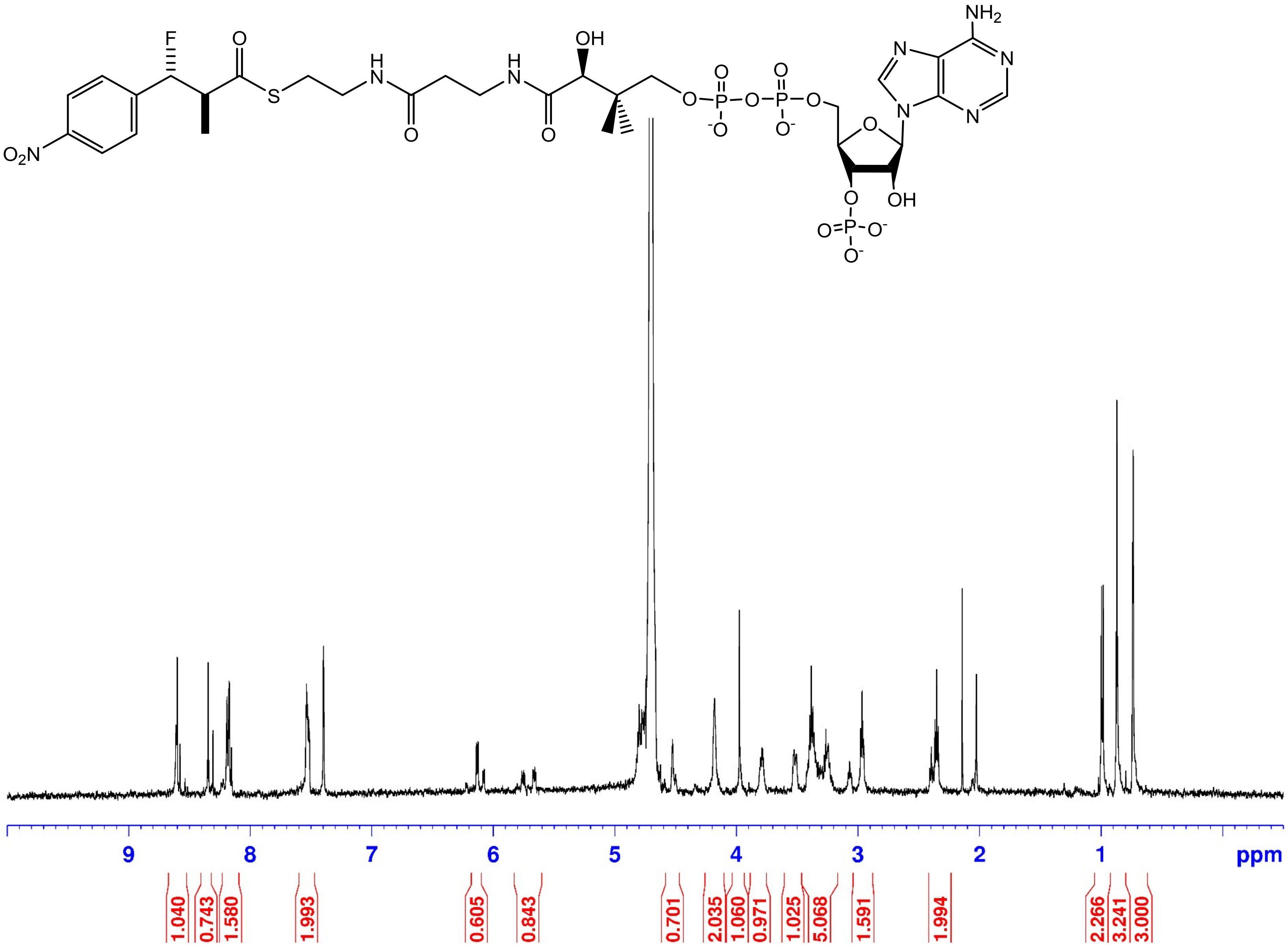

Figure S21. ^{19}F NMR spectrum *anti*-3-Fluoro-2-methyl-3-(4-nitrophenyl)propanoyl-CoA (**30**)

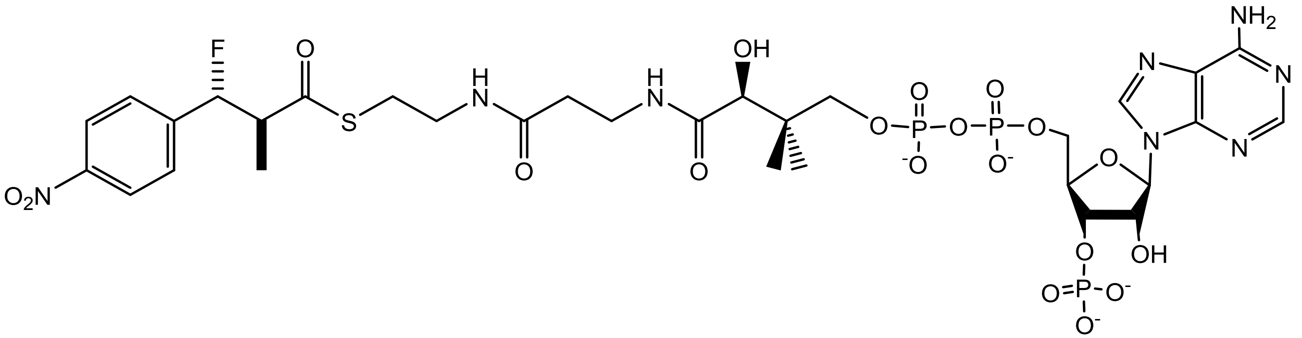

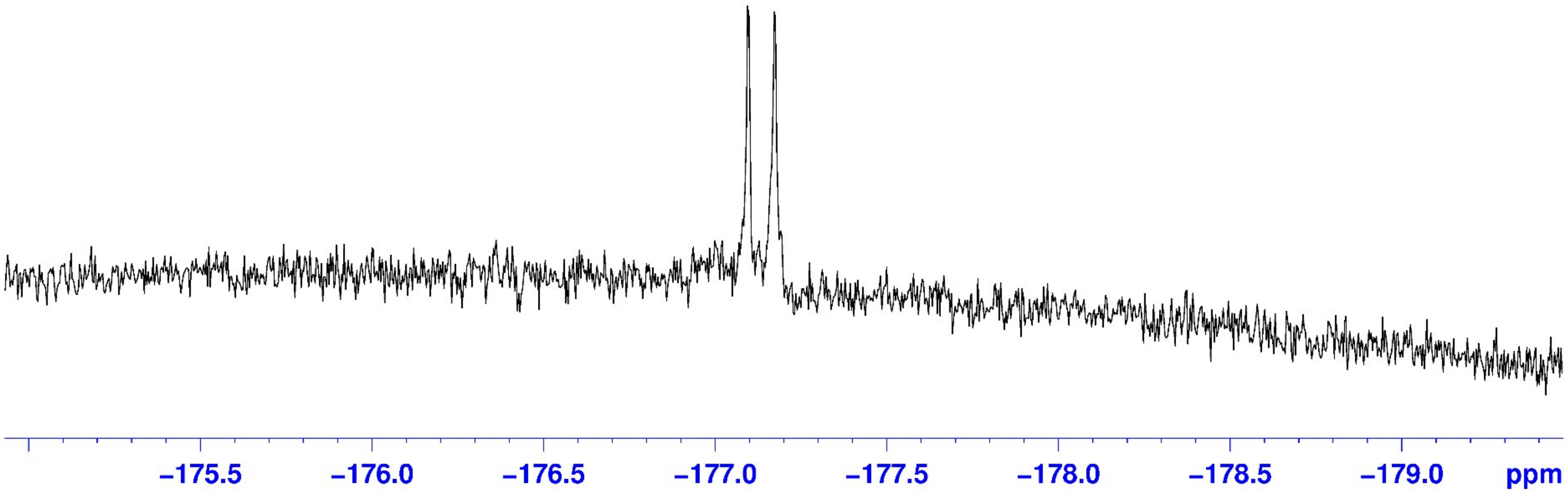

Figure S22. ^{1}H NMR spectrum of 2-(2-Hydroxyphenyl)-2,3-dihydroquinolin-4(1*H*)-one (**35**)

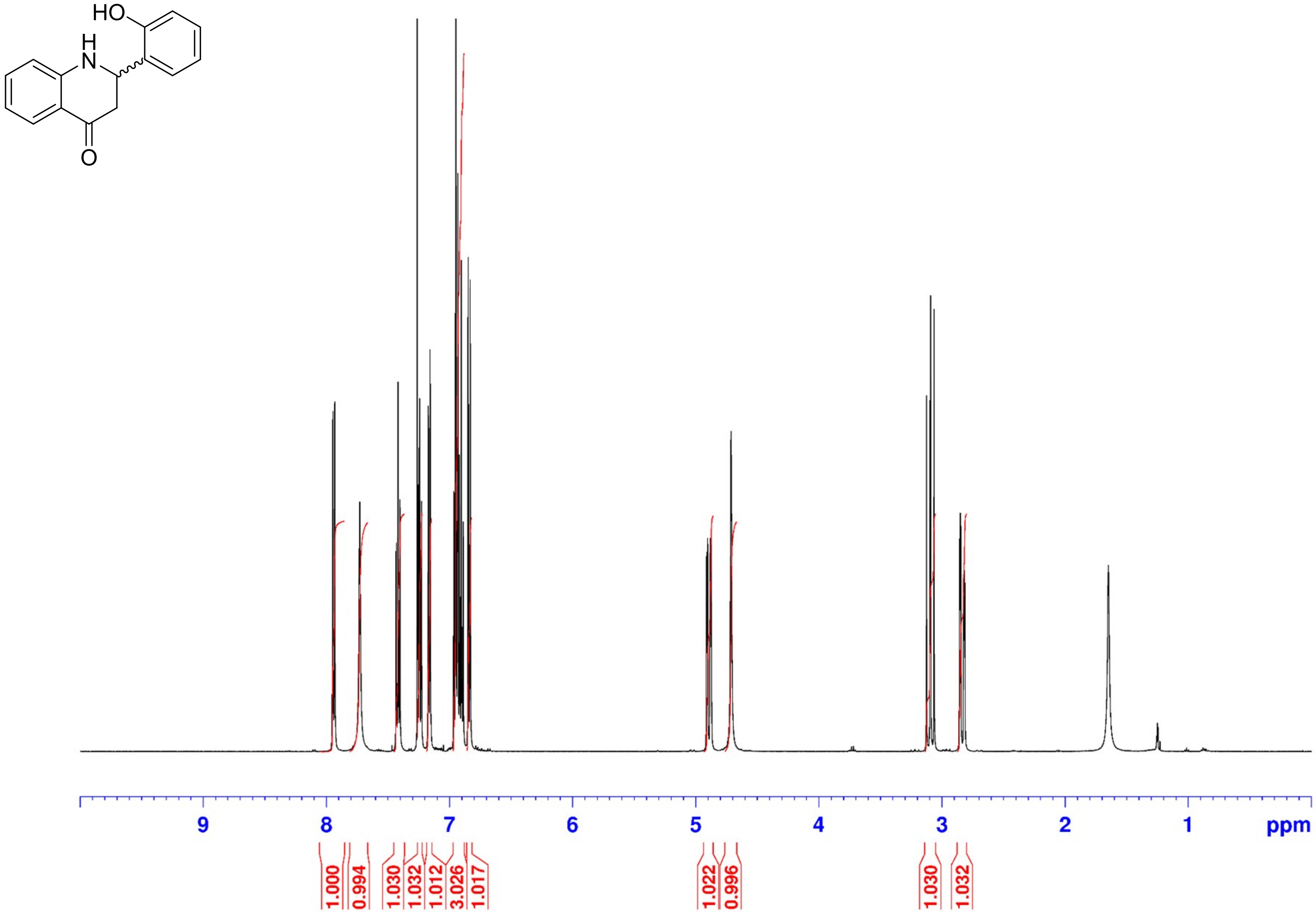

Figure S23. ^{13}C NMR spectrum of 2-(2-Hydroxyphenyl)-2,3-dihydroquinolin-4(1*H*)-one (**35**)

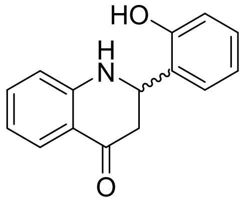

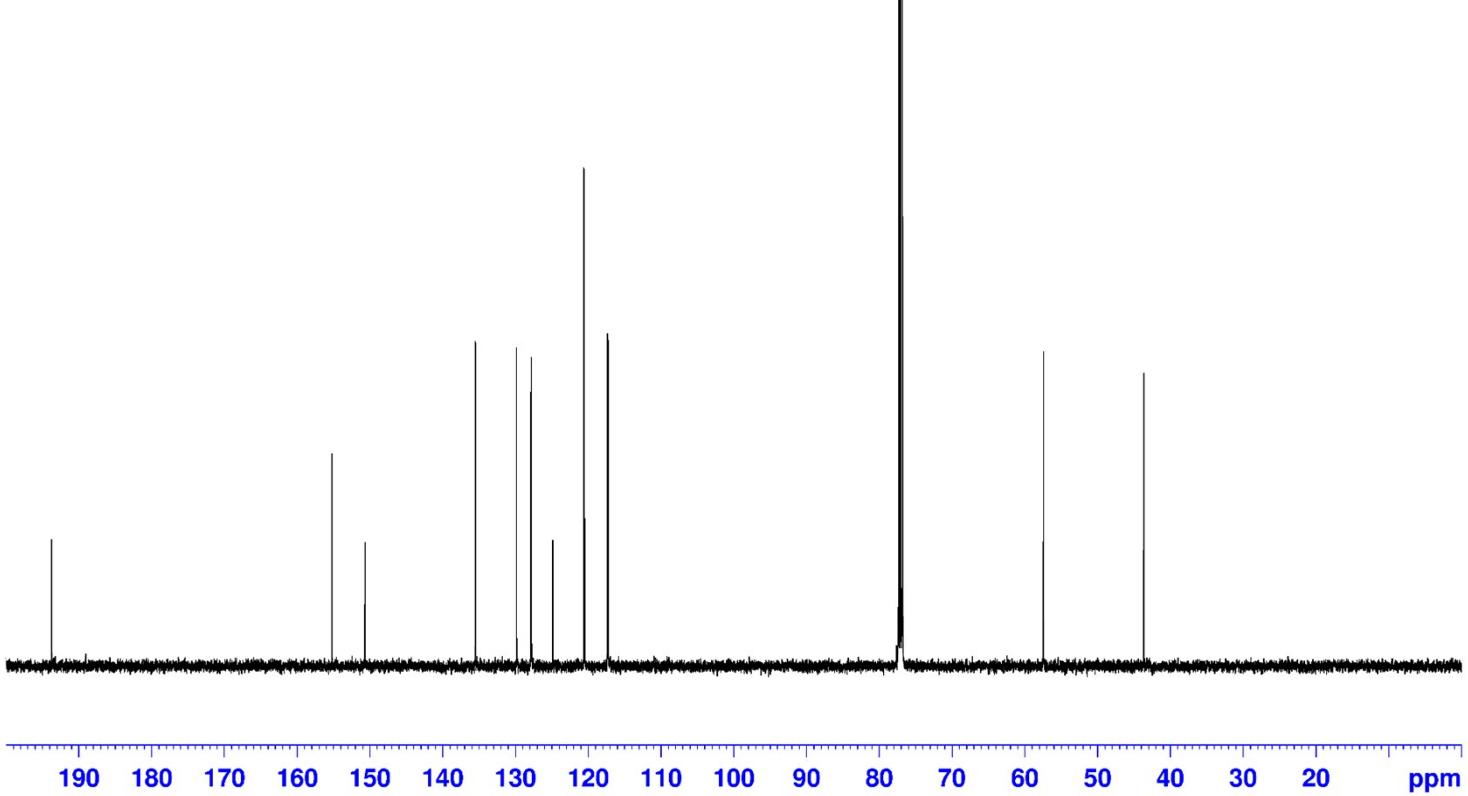

Single Crystal X-Ray Crystallography Data

Single crystals of 2-(2-Hydroxyphenyl)-2,3-dihydroquinolin-4(1H)-one (**35**) were produced by dissolving hot ethanol followed by addition of petroleum ether. A crystal was mounted in inert oil and transferred to the cold gas stream of a Nonius Kappa CCD diffractometer. Intensity data were collected with MoKα radiation (λ = 0.71073 Å) at T = 150(2) K. Data were processed using the Nonius Software.[1] A symmetry related (multi-scan) absorption correction had been applied. The structure was solved with Sir97[2] followed by full-matrix least squares refinement on F^2 using SHELXL-2013[3] implemented in the WINGX-1.80 suite of programmes throughout.[4] Crystal parameters and details on data collection, solution and refinement for the complexes are provided in Table S1.

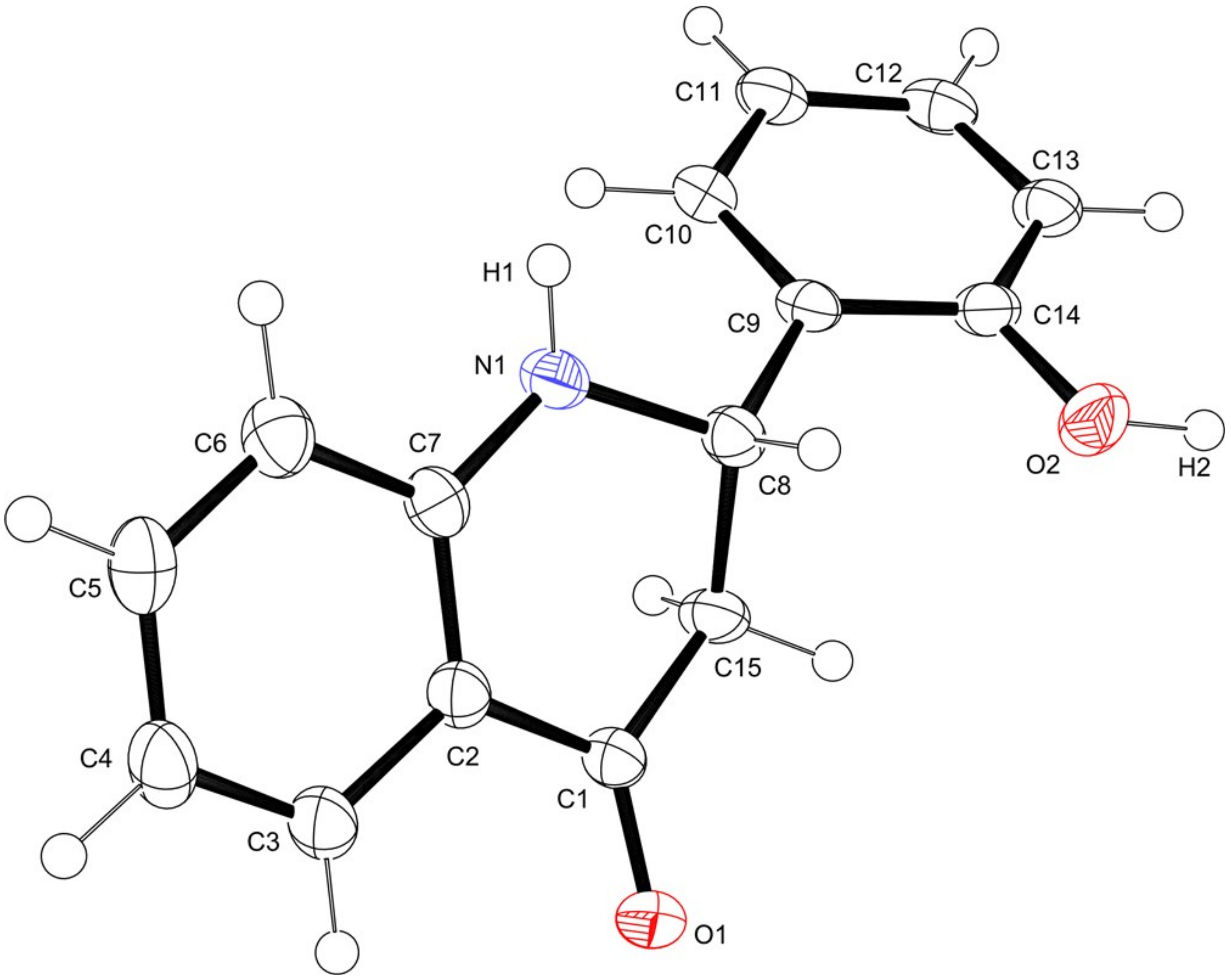

Figure S24. X-Ray crystal structure of 2-(2-Hydroxyphenyl)-2,3-dihydroquinolin-4(1H)-one (**35**) with ellipsoids shown at 50 % probability

Table S1. Crystal data and structure refinement for2-(2-Hydroxyphenyl)-2,3-dihydroquinolin-4(1*H*)-one (**35**).

X-Ray Parameters

Empirical formula	$C_{15} H_{13} N O_2$	
Formula weight	239.26	
Temperature	150(2) K	
Wavelength	0.71073 Å	
Crystal system	Triclinic	
Space group	P -1	
Unit cell dimensions	a = 7.9496(2) Å	α = 108.9192(9)°.
	b = 8.5205(2) Å	β = 98.7190(10)°.
	c = 9.6732(2) Å	γ = 103.0474(10)°.
Volume	585.57(2) $Å^3$	
Z	2	
Density (calculated)	1.357 Mg/m^3	
Absorption coefficient	0.091 mm^{-1}	
F(000)	252	
Crystal size	0.600 x 0.400 x 0.300 mm^3	
Theta range for data collection	3.732 to 30.062°.	
Index ranges	-11<=h<=10, -11<=k<=11, -13<=l<=13	
Reflections collected	13540	
Independent reflections	3395 [R(int) = 0.0467]	
Completeness to theta = 25.242°	99.5 %	
Absorption correction	Semi-empirical from equivalents	
Max. and min. transmission	0.975 and 0.793	
Refinement method	Full-matrix least-squares on F^2	
Data / restraints / parameters	3395 / 0 / 171	
Goodness-of-fit on F^2	1.063	
Final R indices [I>2sigma(I)]	R1 = 0.0463, wR2 = 0.1216	
R indices (all data)	R1 = 0.0640, wR2 = 0.1323	
Extinction coefficient	n/a	
Largest diff. peak and hole	0.270 and -0.250 e.$Å^{-3}$	

Table S2. Bond lengths [Å] and angles [°] for **35**.

N(1)-C(7)	1.3755(14)
N(1)-C(8)	1.4566(14)
N(1)-H(1)	0.884(17)
O(1)-C(1)	1.2341(14)
O(2)-C(14)	1.3608(14)
O(2)-H(2)	0.95(2)
C(1)-C(2)	1.4588(16)
C(1)-C(15)	1.5093(14)
C(2)-C(3)	1.4068(15)
C(2)-C(7)	1.4072(16)
C(3)-C(4)	1.3736(18)
C(3)-H(3)	0.9500
C(4)-C(5)	1.4021(19)
C(4)-H(4)	0.9500
C(5)-C(6)	1.3751(17)
C(5)-H(5)	0.9500
C(6)-C(7)	1.4101(16)
C(6)-H(6)	0.9500
C(8)-C(9)	1.5137(14)
C(8)-C(15)	1.5332(15)
C(8)-H(8)	1.0000
C(9)-C(10)	1.3906(15)
C(9)-C(14)	1.3995(15)
C(10)-C(11)	1.3913(16)
C(10)-H(10)	0.9500
C(11)-C(12)	1.3870(17)
C(11)-H(11)	0.9500
C(12)-C(13)	1.3892(17)
C(12)-H(12)	0.9500
C(13)-C(14)	1.3983(15)
C(13)-H(13)	0.9500
C(15)-H(15A)	0.9900
C(15)-H(15B)	0.9900

Table S3. Bond angles [°] for **35**.

C(7)-N(1)-C(8)	120.02(9)
C(7)-N(1)-H(1)	113.9(10)
C(8)-N(1)-H(1)	115.1(10)
C(14)-O(2)-H(2)	110.7(11)
O(1)-C(1)-C(2)	122.52(10)
O(1)-C(1)-C(15)	121.62(10)
C(2)-C(1)-C(15)	115.76(9)
C(3)-C(2)-C(7)	119.93(10)
C(3)-C(2)-C(1)	120.32(10)
C(7)-C(2)-C(1)	119.73(9)
C(4)-C(3)-C(2)	120.94(12)
C(4)-C(3)-H(3)	119.5
C(2)-C(3)-H(3)	119.5
C(3)-C(4)-C(5)	118.99(11)
C(3)-C(4)-H(4)	120.5
C(5)-C(4)-H(4)	120.5
C(6)-C(5)-C(4)	121.22(11)
C(6)-C(5)-H(5)	119.4
C(4)-C(5)-H(5)	119.4
C(5)-C(6)-C(7)	120.42(11)
C(5)-C(6)-H(6)	119.8
C(7)-C(6)-H(6)	119.8
N(1)-C(7)-C(2)	121.70(10)
N(1)-C(7)-C(6)	119.84(10)
C(2)-C(7)-C(6)	118.42(10)
N(1)-C(8)-C(9)	111.15(9)
N(1)-C(8)-C(15)	108.70(9)
C(9)-C(8)-C(15)	109.39(9)
N(1)-C(8)-H(8)	109.2
C(9)-C(8)-H(8)	109.2
C(15)-C(8)-H(8)	109.2
C(10)-C(9)-C(14)	118.59(9)
C(10)-C(9)-C(8)	122.25(10)
C(14)-C(9)-C(8)	119.05(10)
C(9)-C(10)-C(11)	121.42(10)
C(9)-C(10)-H(10)	119.3
C(11)-C(10)-H(10)	119.3
C(12)-C(11)-C(10)	119.53(11)

C(12)-C(11)-H(11)	120.2
C(10)-C(11)-H(11)	120.2
C(11)-C(12)-C(13)	120.03(10)
C(11)-C(12)-H(12)	120.0
C(13)-C(12)-H(12)	120.0
C(12)-C(13)-C(14)	120.16(11)
C(12)-C(13)-H(13)	119.9
C(14)-C(13)-H(13)	119.9
O(2)-C(14)-C(13)	122.53(10)
O(2)-C(14)-C(9)	117.28(9)
C(13)-C(14)-C(9)	120.19(10)
C(1)-C(15)-C(8)	113.40(9)
C(1)-C(15)-H(15A)	108.9
C(8)-C(15)-H(15A)	108.9
C(1)-C(15)-H(15B)	108.9
C(8)-C(15)-H(15B)	108.9
H(15A)-C(15)-H(15B)	107.7

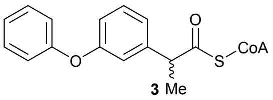

3

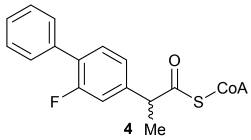

4

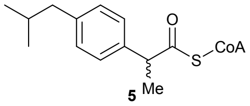

5

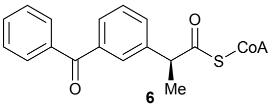

6

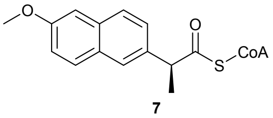

7

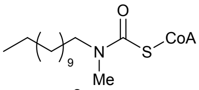

8

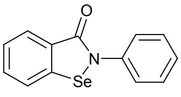

9

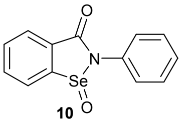

10

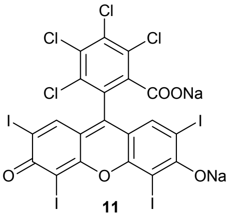

11

Inhibitor	Substrate conversion in positive control	Substrate conversion in presence of inhibitor	Reduction caused by inhibitor	Average reduction caused by inhibitor
**3**	56.2%	42.3%; 41.9%	13.9%; 14.3%	14.10 ± 0.098%
**4**	42.2%	38.1%; 39.2%	4.1%; 3.0%	3.55 ± 0.029%
**5**	56.2%	44.5%; 38.8%	11.7%; 17.4%	14.55 ± 0.082%
**6**	56.2%	55.2%; 48.3%	1.0%; 7.9%	4.45 ± 0.007%
**7**	42.2%	36.2%; 38.6%	6.0%; 3.6%	4.80 ± 0.042%
**8**	56.2%	8.7%; 10.2%	47.5%; 46.0%	46.75 ± 0.335%
**9**	42.2%	-0.1%; -1.1%	42.3%; 43.3%	42.80 ± 0.299%
**10**	45.4%	38.3%; 41.8%	7.1%; 3.6%	5.35 ± 0.050%
**11**	45.4%	0.5%; 0.4%	44.9%; 45.0%	44.95 ± 0.317%

Table S1. Inhibition of AMACR activity by various known drugs. Substrate (3*R*,2*R*-3-fluoro-2-methyldecanoyl-CoA **1**) was at a final concentration of 100 µM and enzyme at a final concentration of 60 µg mL^{-1} (1.27 nM). Average reduction caused by inhibitor is mean ± standards deviation of the sample (calculated using Excel 2013).

Fluorescence Data

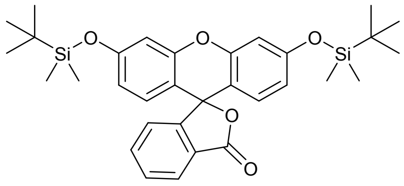

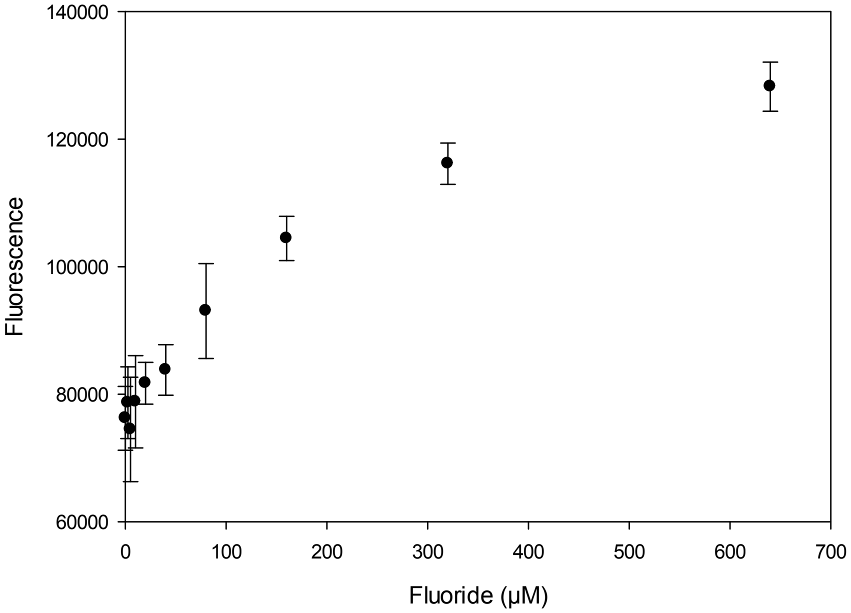

Figure S25: Titration of fluoride (from sodium fluoride) in 50 mM Tris-HCl, pH 7.4 with sensor **33** in DMSO [1:4 (v/v)]. Concentrations of fluoride are for during the incubation with sensor **33** before addition of HEPES-NaOH and fluorescence measurement. The concentration of sensor **33** during the incubation with fluoride was 256 µM. Readings are average of 12 replicants ± standard deviation of the population.

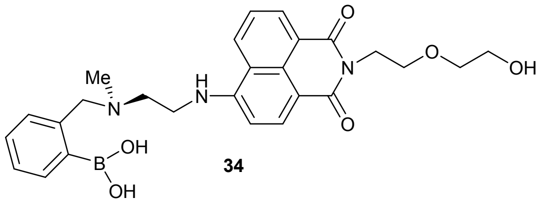

34

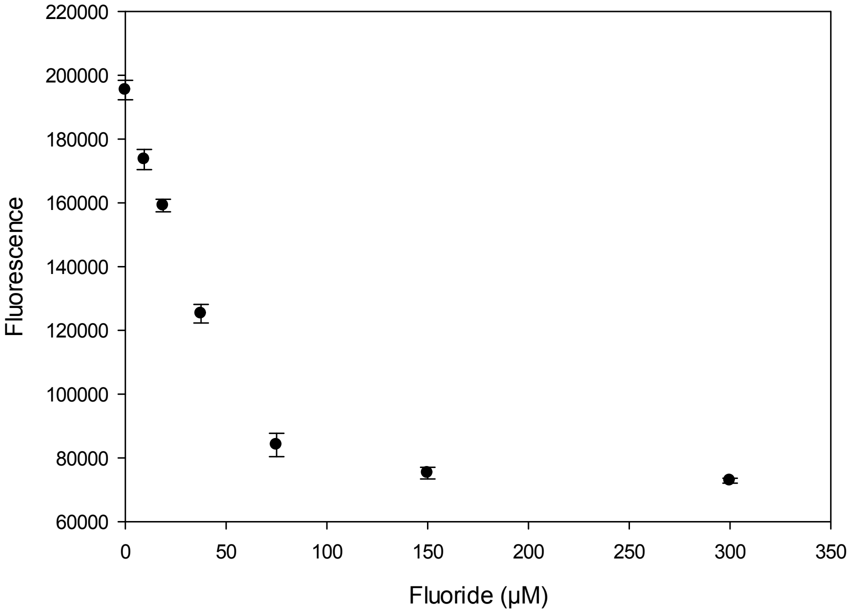

Figure S26: Titration of fluoride (from tert-n-butylammonium fluoride) with sensor **34** in 100% acetonitrile. Concentrations of fluoride are for during the incubation with sensor **34**. The concentration of sensor **34** during incubation with fluoride was 2 µM. Readings are average of 6 replicants ± standard deviation of the population.

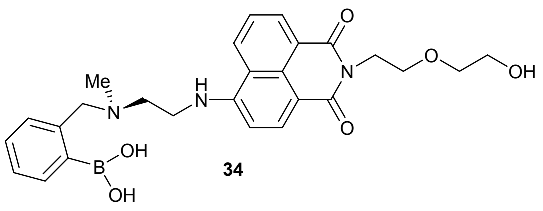

34

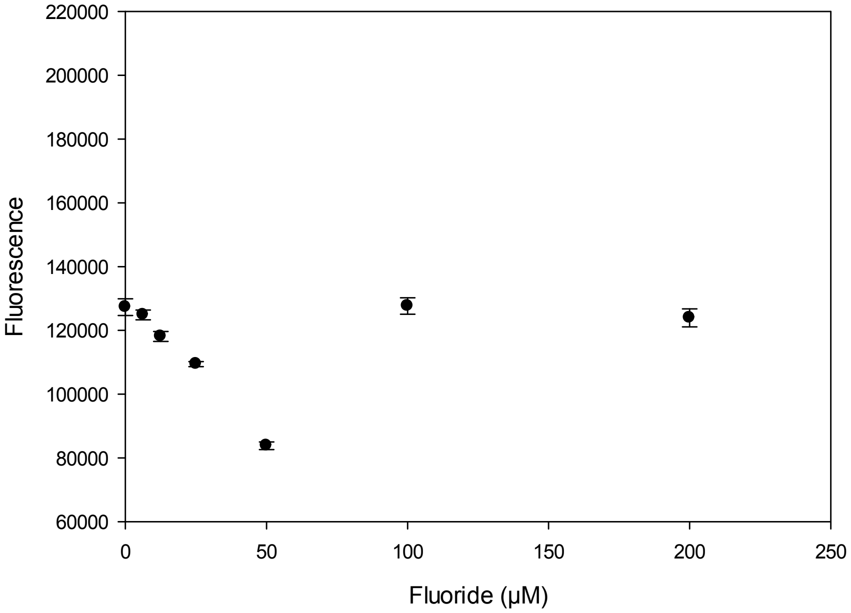

Figure S27: Titration of fluoride (from sodium fluoride) with sensor **34** in 2.5 mM NaH_2PO_4-NaOH, pH 7.4 in acetonitrile/H_2O [19:1 (v/v)]. Concentrations of fluoride are for during the incubation with sensor **34**. The concentration of sensor **34** during incubation with fluoride was 2 μM. Readings are average of 6 replicants ± standard deviation of the population.

References

1. Z. Otwinowski and W. Minor, in *Methods in Enzymology*, *Macromolecular Crystallography, Pt A,* 1997, **276**, pp. 307-326, Academic Press.
2. Altomare, M. C. Burla, M. Camalli, G. L. Cascarano, C. Giacovazzo, A. Guagliardi, A. G. G. Moliterni, G. Polidori and R. Spagna, *J. Appl. Cryst.*, 1999, **32**, 115-119.
3. G. M. Sheldrick, *Acta Cryst. Section A*, 2008, **64**, 112-122.
4. L. J. Farrugia, *J. Appl. Cryst.*, 1999, **32**, 837-838.
